# Nanoscale Resist‐Free Patterning of Halogenated Zeolitic Imidazolate Frameworks by Extreme UV Lithography

**DOI:** 10.1002/advs.202415804

**Published:** 2025-03-05

**Authors:** Weina Li, Tianlei Ma, Pengyi Tang, Yunhong Luo, Hui Zhang, Jun Zhao, Rob Ameloot, Min Tu

**Affiliations:** ^1^ State Key Laboratory of Transducer Technology Shanghai Institute of Microsystem and Information Technology Chinese Academy of Sciences Shanghai 200050 China; ^2^ Center of Materials Science and Optoelectronics Engineering University of Chinese Academy of Sciences Beijing 100049 China; ^3^ 2020 X‐Lab Shanghai Institute of Microsystem and Information Technology Chinese Academy of Sciences Shanghai 200050 China; ^4^ School of Graduate Study University of Chinese Academy of Sciences Beijing 100049 China; ^5^ ShanghaiTech University School of physical science and technology Shanghai 201210 China; ^6^ Shanghai Synchrotron Radiation Facility Shanghai Advanced Research Institute Chinese Academy of Sciences Shanghai 201204 China; ^7^ National Key Laboratory of Materials for Integrated Circuits Shanghai Institute of Microsystem and Information Technology Chinese Academy of Sciences Shanghai 200050 China; ^8^ Centre for Membrane Separations Adsorption Catalysis and Spectroscopy KU Leuven Leuven 3001 Belgium

**Keywords:** EUV lithography, metal–organic frameworks, resist‐free patterning, thin films, zeolitic imidazolate frameworks

## Abstract

Advancements in patterning techniques for metal–organic frameworks (MOFs) are crucial for their integration into microelectronics. However, achieving precise nanoscale control of MOF structures remains challenging. In this work, a resist‐free method for patterning MOFs is demonstrated using extreme ultraviolet (EUV) lithography with a resolution of 40 nm. The role of halogen atoms in the linker and the effect of humidity are analyzed through in situ and near‐ambient pressure synchrotron X‐ray photoelectron spectroscopy. In addition to facilitating the integration of MOFs, the results offer valuable insights for developing the highly sought‐after positive‐tone EUV photoresists.

## Graphical abstract

This work presents a direct, resist‐free patterning method for metal–organic frameworks using extreme ultraviolet (EUV) lithography, achieving a resolution of 40 nm. The EUV mechanism is investigated through synchrotron in situ X‐ray photoelectron spectroscopy. These findings enable the integration of MOFs into electronic devices and provide valuable insights for the development of positive‐tone EUV photoresists.

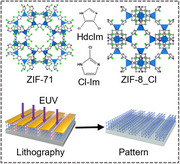

## 1 Introduction

Lithography patterning is a crucial step in integrating new functional materials into electronic applications.^[^ ^1^, ^2^, ^3^, ^4^ ^]^ Metal–organic frameworks (MOFs) have the potential to introduce novel functionalities in microelectronics and optoelectronics, such as low‐k dielectrics, chemical sensors, and field‐effect transistors.^[^ ^5^, ^6^, ^7^, ^8^, ^9^ ^]^ MOF patterning methods can be divided into bottom‐up and top‐down approaches. The bottom‐up approach involves the direct growth of MOFs at well‐designed locations on a substrate, for instance, via microcontact printing of patterned self‐assembled monolayers followed by area‐selective MOF growth.^[^ ^10^, ^11^, ^12^, ^13^, ^14^, ^15^ ^]^ However, achieving high‐resolution (sub‐µm) patterns and sharp edges with this technique is challenging, and non‐selective growth occurs for thicker MOF films. In contrast, top‐down lithographic patterning involves transferring a pattern onto a continuous film, making it more compatible with established industrial fabrication processes.^[^ ^12^, ^13^, ^14^, ^15^, ^16^ ^]^ Typically, this process uses a photosensitive resist layer as an etch mask, which must be deposited, patterned, and removed without altering the properties of the underlying material. Given this, direct, resist‐free lithography of functional materials is highly desirable, as it minimizes process steps and reduces potential contamination sources—especially for MOFs, which are susceptible to pore clogging.^[^ ^17^, ^18^, ^19^, ^20^, ^21^, ^22^, ^23^, ^24^, ^25^, ^26^ ^]^

Recent advancements have demonstrated that certain MOFs can be directly patterned with high resolution using electron‐beam lithography (EBL).^[^ ^27^, ^28^, ^29^, ^30^ ^]^ Tsapatsis et al. achieved sub‐100 nm, resist‐free EBL patterning of zeolitic imidazolate framework (ZIF) films, where the exposed ZIFs functioned as either a positive‐ or negative‐tone resist, depending on the exposure dose and solvent used during developing.^[^ ^27^ ^]^ We have achieved sub‐50 nm resolution in direct EBL patterning of halogenated ZIFs, where e‐beam irradiation induces structural and chemical changes driven by halogen radicals.^[^ ^29^ ^]^ While these resist‐free approaches enable nanoscale patterning of MOFs, EBL is a mask‐less, low‐throughput method, making it suitable only for niche applications, such as mask fabrication and prototyping.

Extreme UV (EUV) lithography is a mask‐based, parallel process compared with EBL, making it well‐suited for mass production in high‐volume semiconductor fabrication. In EUV lithography, high‐energy EUV photons with a wavelength of 13.5 nm and an energy of 92 eV are absorbed by the resist material. This absorption causes electrons to be ejected with energies between 75 and 82 eV, leaving positively charged radicals in the resist layer.^[^ ^31^, ^32^, ^33^, ^34^ ^]^ These EUV‐generated electrons and holes trigger chemical reactions, such as bond cleavage and cross‐linking, which alter the solubility of the photoresist.^[^ ^35^, ^36^, ^37^, ^38^ ^]^

The motivation to explore EUV patterning of MOFs is twofold. On the one hand, EUV lithography offers a mask‐based and scalable method for integrating the unique properties of MOFs into electronic devices. Since the photoelectrons generated during EUV exposure trigger the solubility change in the resist, MOF materials that can be patterned with EBL may also be suitable for EUV lithography. On the other hand, there is an urgent need to develop novel photoresists tailored for EUV lithography. Conventional organic photoresists, primarily composed of light elements, have limited interaction with high‐energy EUV photons, resulting in low exposure sensitivity. Consequently, metal‐containing resists are being explored to achieve a higher EUV absorption cross‐section.^[^ ^31^, ^34^, ^39^, ^40^, ^41^ ^]^ However, these resists are almost exclusively negative‐tone, meaning that the pattern is created by retaining the exposed areas after development. This reliance on negative‐tone photoresists poses challenges for processes that require positive‐tone materials, such as contact‐hole patterning in memory devices. Given that some MOFs have demonstrated positive‐tone patterning in EBL, investigating these materials as positive EUV photoresists presents an exciting opportunity.

**Figure 1:**
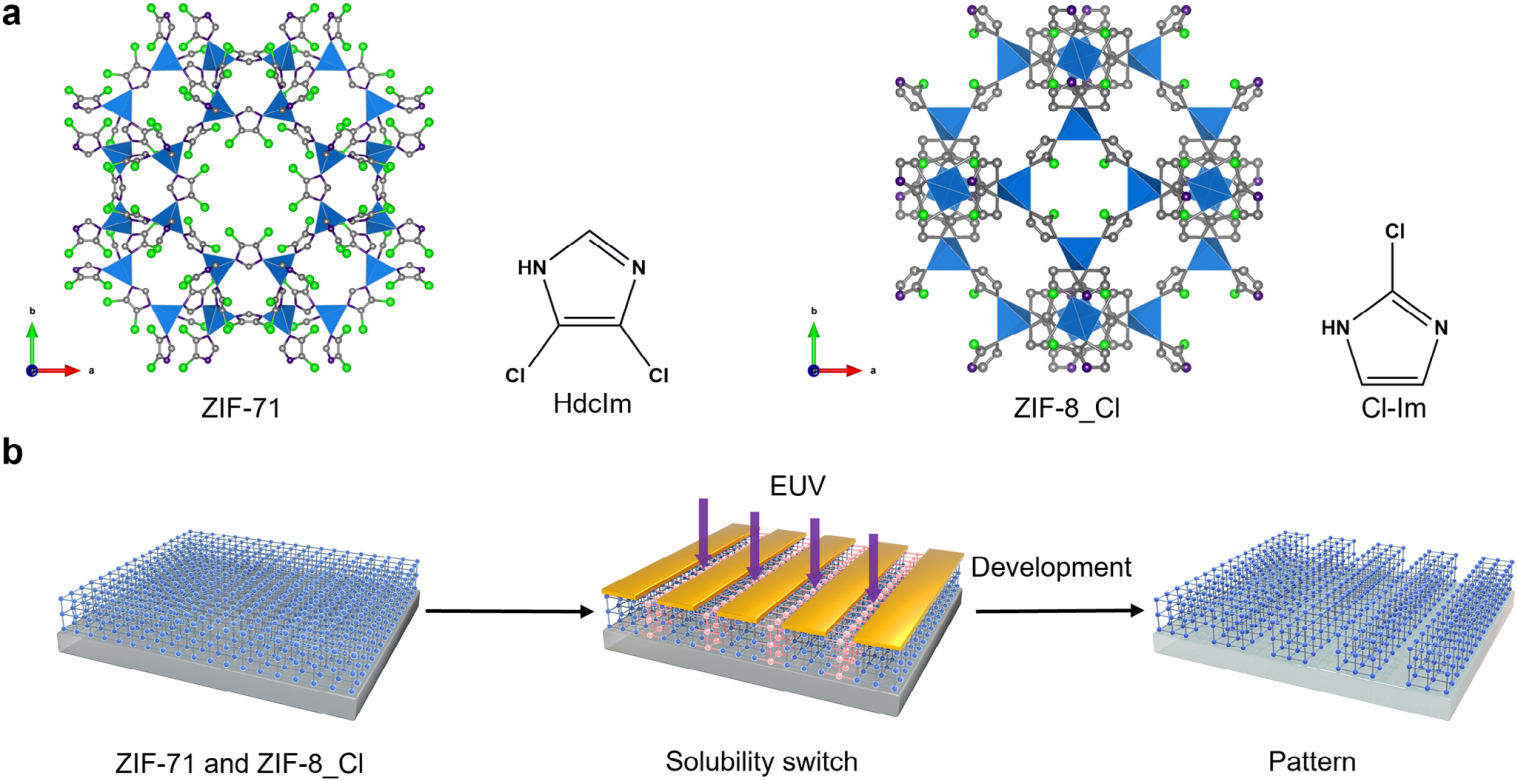
Direct EUV lithographic patterning of halogenated ZIF films. a) Representation of the crystal structures of ZIF‐71 and ZIF‐8_Cl and the corresponding linkers 4,5‐dichloroimidazole (HdcIm) and 2‐chloroimidazole (Cl‐Im). b) Schematic illustration of the resist‐free EUV lithography of ZIF films, which behave as positive‐tone resists.

In this study, we report the direct, resist‐free patterning of halogenated ZIFs using EUV lithography, achieving a resolution down to 40 nm (**Figure** **1**). Additionally, we examined the chemical changes in the halogenated ZIF thin film under EUV irradiation using in situ synchrotron X‐ray photoelectron spectroscopy (XPS). We also explored the impact of humidity on the EUV sensitivity of halogenated ZIFs through synchrotron near‐ambient XPS.

## 2 Results and Discussion

**Figure 2:**
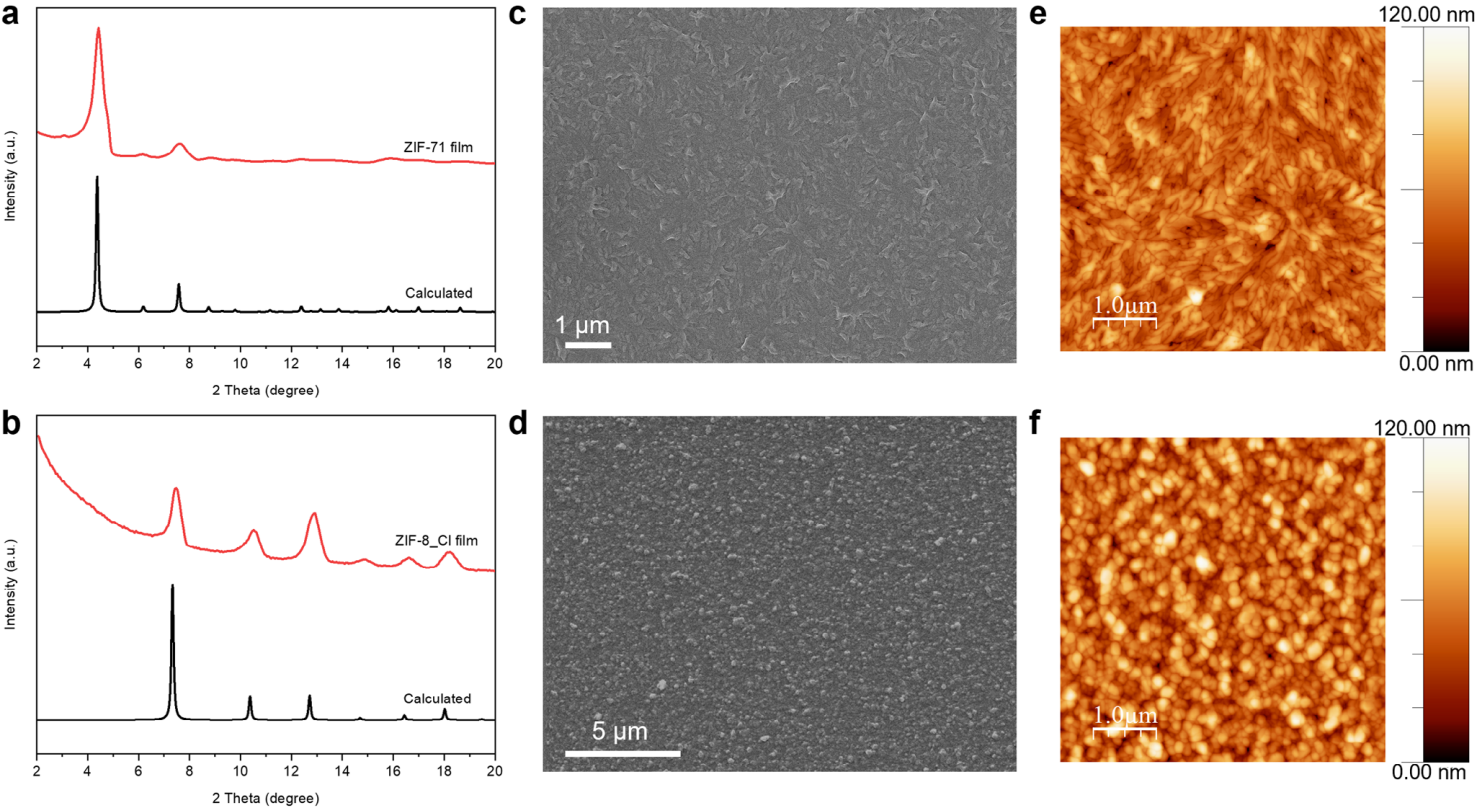
Characterization of halogenated ZIF films. a, b) GIXRD of ZIF‐71 and ZIF‐8_Cl films. c, d) SEM images of ZIF‐71 and ZIF‐8_Cl films. e, f) AFM images of ZIF‐71 and ZIF‐8_Cl films.

Lithographic patterning requires smooth, thin films. To meet these requirements, we use chemical vapor deposition (CVD) to grow halogenated ZIF thin films.^[^ ^29^, ^42^, ^43^ ^]^ ZIF‐71 and ZIF‐8_Cl are synthesized by coordinating Zn^2+^ with 4,5‐dichloroimidazole (HdcIm) and 2‐chloroimidazole (Cl‐Im), respectively, forming RHO and SOD topologies.^[^ ^44^, ^45^ ^]^ Thin films of these MOFs are produced by first depositing ZnO films via atomic layer deposition (ALD) and then converting the precursor films into an atmosphere containing the corresponding linkers and template vapors.^[^ ^42^ ^]^ The grazing incidence X‐ray diffraction (GIXRD) peaks of the resulting ZIF‐71 and ZIF‐8_Cl films align with the calculated PXRD patterns, confirming the formation of the expected phases (**Figure** **2**a,b). Scanning electron microscopy (SEM) and atomic force microscopy (AFM) images show that uniform and homogeneous ZIF‐71 and ZIF‐8_Cl thin films are successfully deposited (Figure 2c–f). The root mean square roughness values for ZIF‐71 and ZIF‐8_Cl thin films are ≈12 and 16.7 nm, respectively. ZIF thin films of different thicknesses can be obtained by converting ZnO films with varying initial thicknesses (Figures S1 and S2, Supporting Information). The theoretical expansion factors for ZIF‐71 and ZIF‐8_Cl films starting from ZnO precursors are 20 and 16.5, respectively.^[^ ^42^ ^]^ ZIF‐71 films with thicknesses of 100 and 60 nm were derived from 5 and 3 nm ZnO layers, respectively, while a ZIF‐8_Cl film with a thickness of 80 nm was derived from a 5 nm ZnO layer.

**Figure 3:**
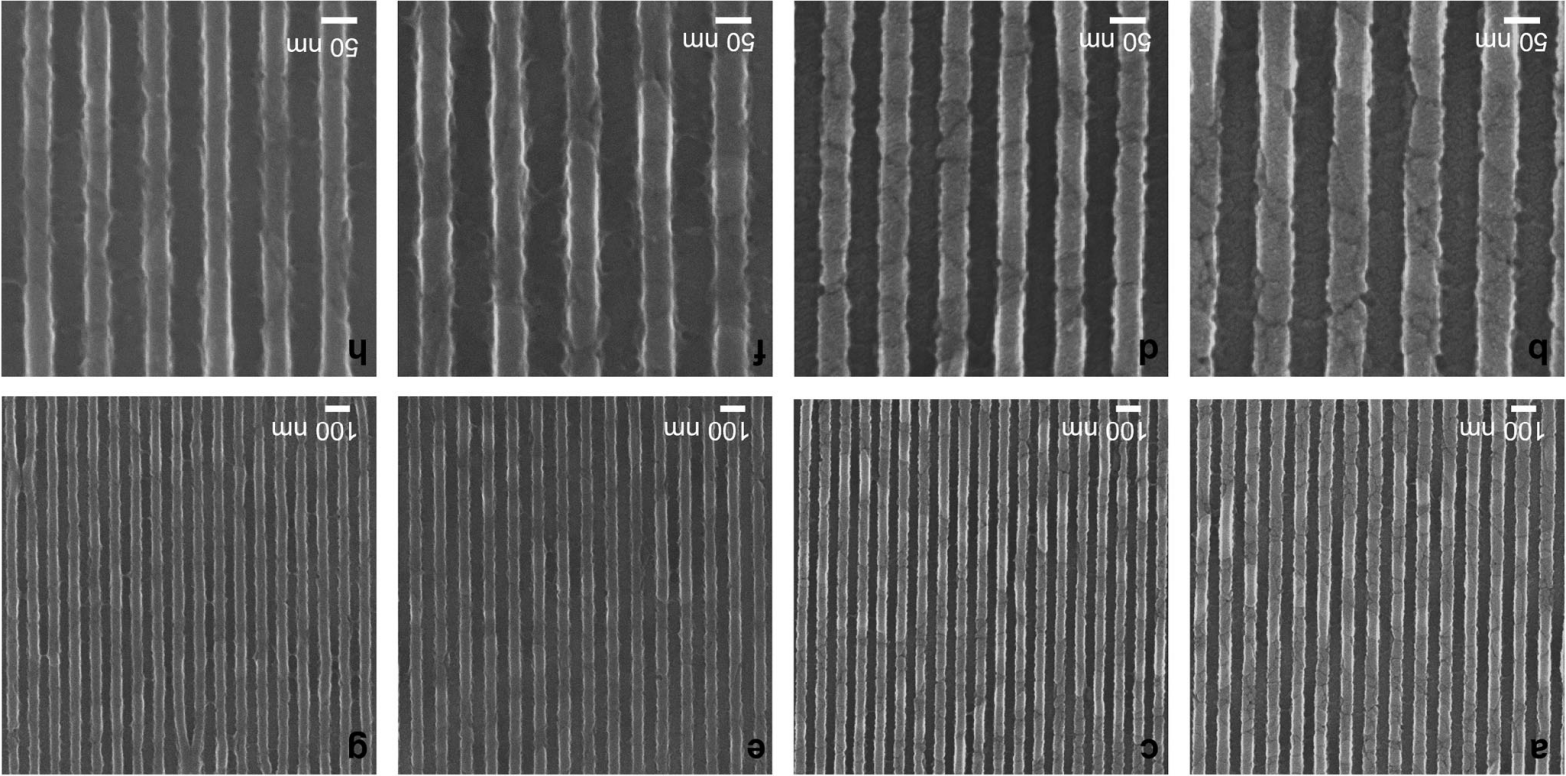
SEM images of ZIF patterns fabricated by EUV lithography. a, b) 50 nm HP pattern from 60 nm ZIF‐71 film under a dose of 440 mJ cm^−2^. c, d) 40 nm HP pattern from 60 nm ZIF‐71 film under a dose of 425 mJ cm^−2^. e, f) 50 nm HP pattern from 80 nm ZIF‐8_Cl film under a dose of 400 mJ cm^−2^. g, h) 40 nm HP pattern from 80 nm ZIF‐8_Cl film under a dose of 265 mJ cm^−2^.

The feasibility of directly patterning halogenated ZIF films using EUV interference lithography was investigated at the Swiss Light Source and the Shanghai Synchrotron Radiation Facility.^[^ ^15^, ^16^, ^17^, ^18^, ^19^, ^20^, ^21^, ^22^, ^23^, ^24^, ^25^, ^26^, ^27^, ^28^, ^29^, ^30^, ^31^, ^32^, ^33^, ^34^, ^35^, ^36^, ^37^, ^38^, ^39^, ^40^, ^41^, ^42^, ^43^, ^44^, ^45^, ^46^ ^]^ The films were exposed to EUV radiation at varying doses and then developed in dimethylsulfoxide (DMSO). Both ZIF‐71 and ZIF‐8_Cl films were found to be amenable to direct patterning through EUV lithography (**Figure** **3**). The contrast curve for the ZIF‐71 film shows a clear step (Figure S3, Supporting Information), with over 90% of the original film being removed at exposure doses above 400 mJ cm⁻^2^. For a 50 nm half‐pitch (HP) pattern, the optimal EUV dose for a 100 nm thick ZIF‐71 film was found to range from 440 to 530 mJ cm^−2^ (Figure S4, Supporting Information). Exposure doses below 440 mJ cm^−2^ led to incomplete development of the exposed regions, while doses above 530 mJ cm^−2^ caused the lines to narrow, increasing the risk of pattern collapse. The optimal exposure dose and resulting pattern quality are strongly dependent on the ZIF film thickness.^[^ ^47^, ^48^ ^]^ Thinner films require lower exposure doses due to the reduced EUV radiation attenuation,^[^ ^49^ ^]^ while thicker films are more prone to pattern collapse at high resolution due to the increased aspect ratio of the formed patterns.^[^ ^47^, ^48^ ^]^ Using an EUV dose of 480 mJ cm⁻^2^, a 50 nm pattern with a line‐edge roughness (LER) of 5.5 nm was achieved in a 100 nm thick ZIF‐71 film (Figure S4, Supporting Information). However, higher resolution patterns (with HPs of 40 and 30 nm) collapsed due to the aspect ratio exceeding 2 (Figures S5 and S6, Supporting Information). To improve pattern quality, a thinner 60 nm ZIF‐71 film was deposited (Figure 3; Figures S7–S11, Supporting Information). The thinner film enabled a 50 nm line with a 1.0 nm LER at an exposure dose of 440 mJ cm^−2^ (Figure 3a,b), and a 40 nm line with an 8.5 nm LER at 425 mJ cm^−2^ (Figure 3c,d). Compared to the 100 nm ZIF‐71 film, the 60 nm film produced higher‐quality patterns with lower LER for 50 nm and smaller features.

**Figure 4:**
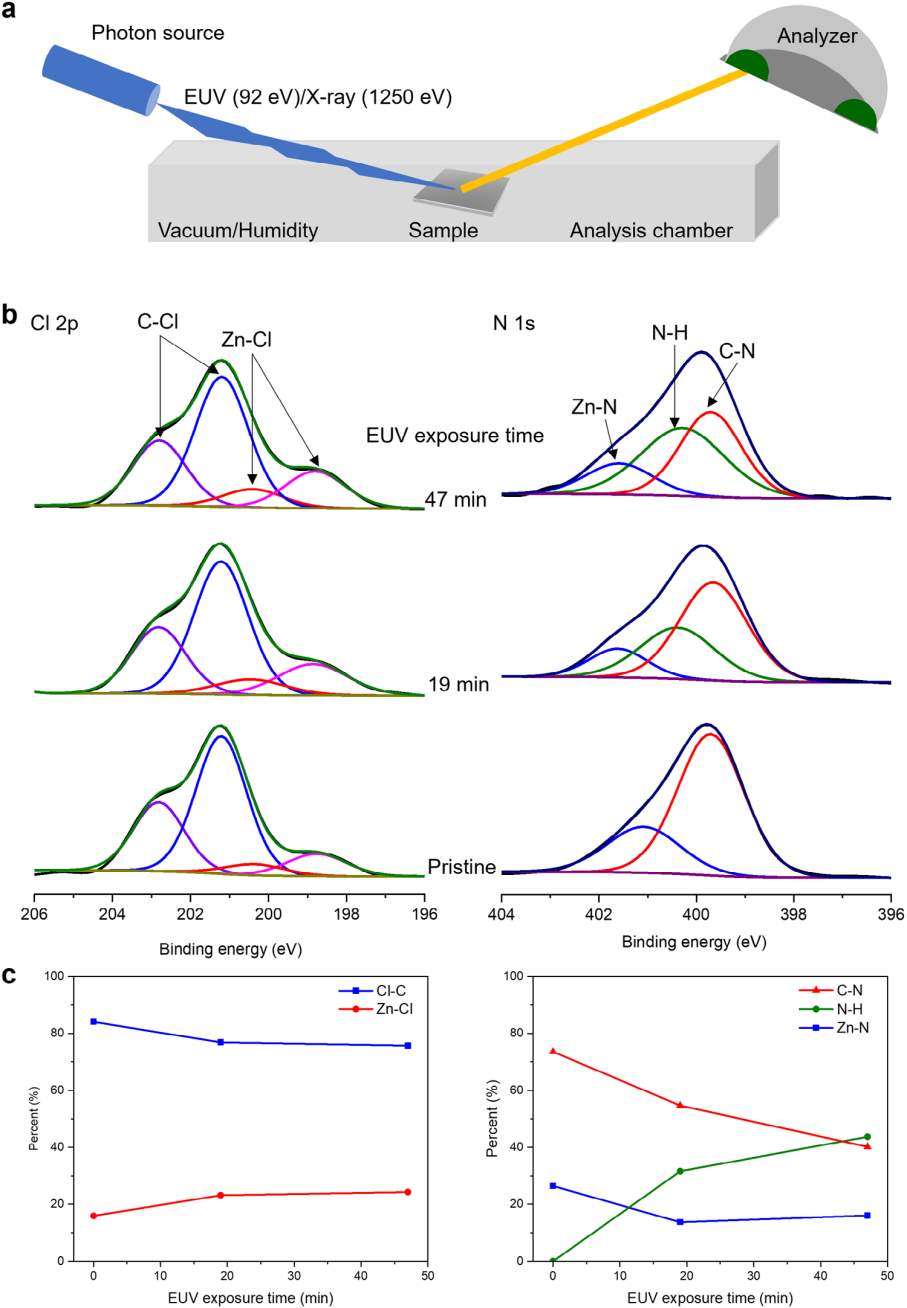
In situ XPS studies of EUV irradiated ZIF‐71 film. a) Schematic illustration of the in situ synchrotron XPS measurement. b) XPS spectra of Cl 2p (left) and N 1s (right) as a function of EUV exposure times. c) The proportion of chlorine bonds (C─Cl and Zn─Cl, left) and nitrogen bonds (Zn─N, N─H, and C─N, right) as a function of EUV exposure time.

We performed in situ synchrotron XPS experiments to investigate the chemical changes in halogenated ZIFs induced by EUV irradiation (**Figure** **4**a). After EUV exposure at 92 eV in a vacuum, XPS spectra were collected from the exposed area using 1250 eV photons. This approach ensures that XPS analysis is conducted directly on the EUV‐exposed region and prevents air exposure during sample transfer, ensuring that any chemical changes in the films are solely attributed to EUV radiation. The XPS spectra of the Zn 2p region showed no significant changes with varying EUV exposure, indicating that Zn^2^⁺ remained unchanged (Figure S16, Supporting Information).^[^ ^50^ ^]^ The fitted Cl 2p spectra revealed two components, corresponding to C─Cl (≈201.2 eV) and Zn─Cl (≈198.9 eV) bonds (Figure 4b).^[^ ^51^ ^]^ The N 1s spectra were fitted with three components, assigned to Zn─N (≈401.6 eV), N─H (≈400.3 eV), and C─N (≈399.7 eV) bonds (Figure 4b).^[^ ^29^, ^52^, ^53^ ^]^ In the Cl 2p region, EUV exposure led to a decrease in the C─Cl bond signal and a corresponding increase in the Zn─Cl bond signal. The presence of Zn─Cl in the pristine ZIF‐71 film was minimal, likely due to beam damage from X‐ray exposure during the measurement. In the N 1s XPS spectra, the Zn─N and C─N signals decreased, while the N─H signal increased (Figure 4b,c).

EUV irradiation can cause both primary and secondary damage to the resist material.^[^ ^32^, ^34^, ^36^, ^54^, ^55^, ^56^, ^57^, ^58^ ^]^ Primary damage occurs when EUV light interacts with the material, leading to bond breaking through photoelectric effects. Secondary damage results from reactions triggered by radiolytic products, such as free radicals generated by energetic electrons. The reduction of the C─Cl bond signal suggests the chlorine radicals are generated by the homolysis of the C─Cl bonds upon the EUV irradiation (Figure 4b,c). These chlorine radicals likely cleave the Zn─N coordination bonds and C─N bonds in the imidazole ring (Figures S17 and S18, Supporting Information).^[^ ^59^, ^60^, ^61^, ^62^ ^]^ Further reactions between the resulting fragments may make the exposed sample soluble in DMSO. While identifying the exact products formed upon EUV irradiation of a thin resist film remains challenging, it is clear the halogen atoms in the linker play a crucial role in the solubility change (Figure S19, Supporting Information).

**Figure 5:**
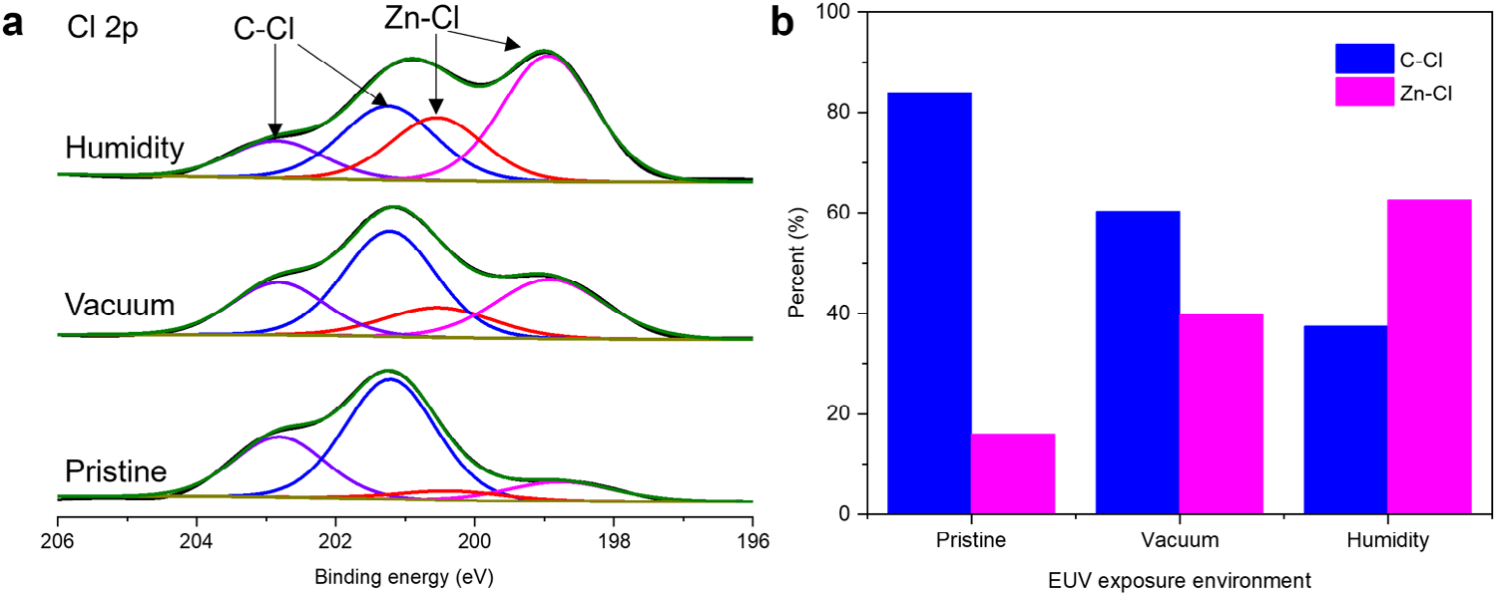
Near‐ambient pressure XPS analysis of humidity effects on EUV‐irradiated ZIF‐71 film. a) XPS spectra of Cl 2p exposed 60 min in different EUV exposure environments (exposure in a vacuum and humidity). b) The proportion of chlorine bonds (C─Cl and Zn─Cl, top) in different EUV exposure environments (exposure in a vacuum and humidity).

EUV lithography is typically conducted in a high vacuum to prevent the EUV photons from interacting with the atmosphere, protect sophisticated optical components, and achieve the highest resolution and pattern quality. However, the development process of EUV resists generally occurs under standard cleanroom conditions. As a result, after exposure to EUV light, the latent image in the resist layer is often exposed to humidity. For other types of EUV resists, it has been reported that this exposure to a cleanroom atmosphere during the delay between EUV exposure and pattern development can impact the critical dimensions that can be achieved.^[^ ^63^ ^]^ We employed synchrotron near‐ambient pressure XPS to further explore the effects of humidity on the chemical changes induced by EUV irradiation. Two scenarios were investigated: 1) the ZIF‐71film was exposed to EUV radiation in a controlled‐humidity atmosphere (**Figure** **5**), and 2) the film was exposed EUV under vacuum, followed by exposure to a humid atmosphere. In the first scenario, we found that exposure to EUV in the presence of humidity led to a higher ratio of Zn─Cl to C─Cl bonds compared to exposure in the vacuum (Figure 5). A similar trend was observed in the ratio of the N─H bond to C─N bonds (Figure S20, Supporting Information). This suggests that the presence of water vapor during exposure may immediately trigger hydrolysis of EUV‐induced products, resulting in a higher Zn─Cl signal. The second scenario showed similar effects (Figure S21, Supporting Information), even when exposure to humidity was delayed, indicating that humidity influences both primary and secondary types of EUV‐induced damage. This finding underscores the importance of controlling the development environment to ensure consistency and reproducibility in EUV lithography.^[^ ^63^ ^]^

## 3 Conclusion

In summary, we demonstrated that halogenated ZIFs (ZIF‐71 and ZIF‐8_Cl) can be directly patterned by EUV lithography, achieving a half‐pitch as small as 40 nm. Further improvements in patterning resolution could be achieved by reducing film thickness and surface roughness. In situ synchrotron XPS studies revealed that EUV exposure leads to the breaking of Zn─N coordination bonds and the ring‐opening of the imidazolate linker. Near‐ambient pressure XPS analysis indicated that humidity accelerates the dissociation of Zn─N coordination bonds and enhances the reactions between EUV‐induced fragments. Our results not only facilitate the integration of MOFs into microelectronics but also provide valuable insights for advancing the development of highly sought‐after positive‐tone EUV photoresists.

## 4 Experimental Section

### Materials

All the reagents were purchased from Sigma‐Aldrich and used without further purification. 4,5‐dichloroimidazole (HdcIm, 97%), 2‐chloroimidazole (Cl‐Im, 97%), N, N‐dimethylformamide (DMF, 99%), dimethyl sulfoxide (DMSO, 99%), acetone (99%), and diethylzinc (DEZ, 95%).

### Deposition of Halogenated ZIF Films: ZIF‐71 and ZIF‐8_Cl

The deposition of the MOF film by CVD involves two steps: 1) deposition of the ZnO film by ALD; 2) vaporized linker reacted with ZnO film in the presence of a template vapor.^[^ ^42^, ^50^, ^64^ ^]^ ZnO films were deposited by a Savannah S‐200 thermal ALD reactor (Veeco Instruments, Inc.) with deionized water and diethylzinc as precursors. The reactant and purge times were set to 0.015 s and 5 s, respectively. The ALD reactor pressure was ≈0.4 mbar and the reaction temperature was at 120 °C. The growth rate of the ZnO film was ≈1.7 Å per cycle. For ZIF‐71 deposition, a ZnO film, 50 mg of HdcIm powder, and 50 µL of DMF were placed separately in a 250 mL Schlenk tube and heated in a convection oven at 110 °C for one day. The obtained ZIF‐71 films were activated under a dynamic vacuum. ZIF‐8_Cl films were deposited by the same process but with Cl‐Im instead of HdcIm.

### Characterization

GIXRD measurements of halogenated ZIF films were conducted using the Malvern PANalytical Empyrean diffractometer, which was equipped with a PIXcel3D solid‐state detector and a Cu anode (λ = 1.5406 Å) operating at 45 kV and 40 mA. These measurements were taken in reflection geometry with an incident beam angle of 0.02°, covering a 2θ range of 2–20°, with a step size of 0.053° and a counting time of 1000 seconds per step. To control beam divergence, a 1/32° fixed anti‐scatter slit was used on the incident beam side. The morphology of the MOF films and patterns were recorded on FEI XL30FEG and Raith E‐line instrument. The thin film samples were sputtered with 5 nm platinum, while the pattern samples were measured directly without coating. ImageJ was used to calculate LER based on SEM images.^[^ ^65^, ^66^ ^]^ AFM images were obtained using a PicoSPM (5100, Agilent Technologies) instrument in intermittent contact mode. The Silicon cantilevers (OLYMPUS, AC160TS‐R3) had a resonance frequency of 300 kHz. Data processing was conducted with Gwyddion 2.44.

### EUV Exposure and Development

EUV lithography was carried out at the XIL‐II Beamline of Swiss Light Source and the Beamline of BL08U1B of the Shanghai Synchrotron Radiation Facility. The pressure in the exposure chamber was ≈10^−7^ mbar. The EUV exposure doses were calculated based on the photo fluxes, exposure times, and areas. After EUV exposure, the MOF film was developed in DMSO for 30 s, rinsed with acetone, and dried using a gentle nitrogen gas stream.

### In Situ and Near‐Ambient Pressure XPS

The in situ and near‐ambient pressure XPS experiments were conducted at the 02B01 beamline of the Shanghai Synchrotron Radiation Facility. Different fresh areas (≈200 × 75 µm^2^) of the ZIF‐71 film were exposed to varying durations at 92 eV in a vacuum (10^−9^ mbar), followed by XPS measurements at 1250 eV. To investigate the effect of water vapor, the ZIF‐71 film was exposed to 92 eV after introducing 1 mbar water vapor, with subsequent XPS measurement at 1250 eV. In another scenario, 1 mbar of water vapor was introduced in the chamber after EUV irradiation, and XPS was measured at the same position. XPS spectra of Cl 2p, N 1s, C 1s, and Zn 2p were acquired. The XPS data were processed by Casa XPS software using the Shirley background and the Gaussisn‐Lorentzian function.

## Conflict of Interest

The authors declare no conflict of interest.

## Supporting information



Supporting Information

## Data Availability

The data that support the findings of this study are available from the corresponding author upon reasonable request.
