# Supplementary material for: Nanoscale Resist‐Free Patterning of Halogenated Zeolitic Imidazolate Frameworks by Extreme UV Lithography
Source: Adv Sci (Weinh). 2025 Mar 5;12(16):2415804. doi: 10.1002/advs.202415804 (PMC12021036; doi:10.1002/advs.202415804)
Supplement: Supplementary file 1 — Supporting Information [file ADVS-12-2415804-s001.docx]

**Supporting Information**

**Nanoscale Resist-free Patterning of Halogenated Zeolitic Imidazolate Frameworks by Extreme UV Lithography**

*Weina Li,^1,2,3,4^ Tianlei Ma,^1,2,3^ Pengyi Tang,^1,2,3,4^ Yunhong Luo,^5^ Hui Zhang,^6,7^Jun Zhao,^6^Rob Ameloot^8*^ and Min Tu^1,2,3,4*^*

^1^State Key Laboratory of Transducer Technology, Shanghai Institute of Microsystem and Information Technology, Chinese Academy of Sciences, Shanghai 200050, China;

^2^Center of Materials Science and Optoelectronics Engineering, University of Chinese Academy of Sciences, Beijing 100049, China.

^3^2020 X-Lab, Shanghai Institute of Microsystem and Information Technology, Chinese Academy of Sciences, Shanghai 200050, China;

^4^School of Graduate Study, University of Chinese Academy of Sciences, Beijing 100049, China;

^5^ShanghaiTech University, School of physical science and technology, Shanghai 201210, China;

^6^Shanghai Synchrotron Radiation Facility, Shanghai Advanced Research Institute, Chinese Academy of Sciences, Shanghai 201204, China;

^7^National Key Laboratory of Materials for Integrated Circuits, Shanghai Institute of Microsystem and Information Technology, Chinese Academy of Sciences, Shanghai 200050, China;

^8^Centre for Membrane Separations, Adsorption, Catalysis and Spectroscopy, KU Leuven, Leuven 3001, Belgium;

*Corresponding authors: [rob.ameloot@kuleuven.be](mailto:rob.ameloot@kuleuven.be); min.tu@mail.sim.ac.cn

**Results and Discussion**

**
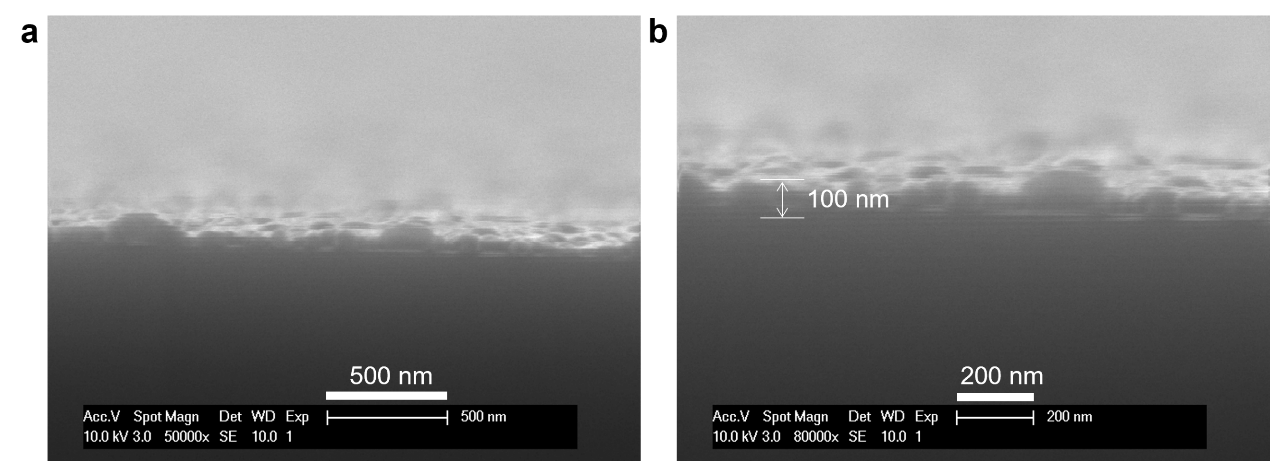
**

**Fig S1.** Cross-section SEM image of 100 nm ZIF-71 film at low (a) and high (b) magnification.


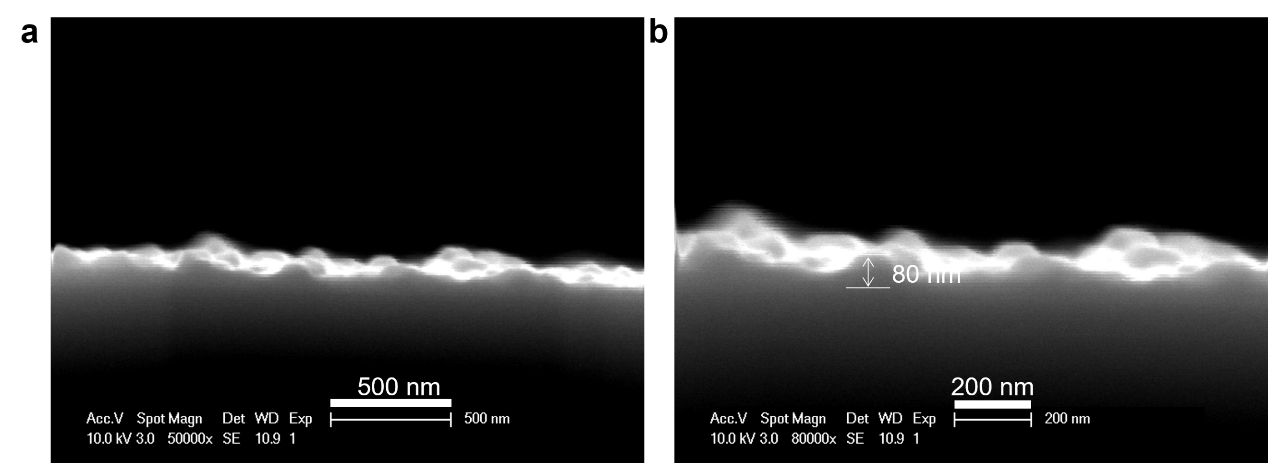


**Figure S2.** Cross-section SEM image of 80 nm ZIF-8_Cl film at low (a) and high (b) magnification.


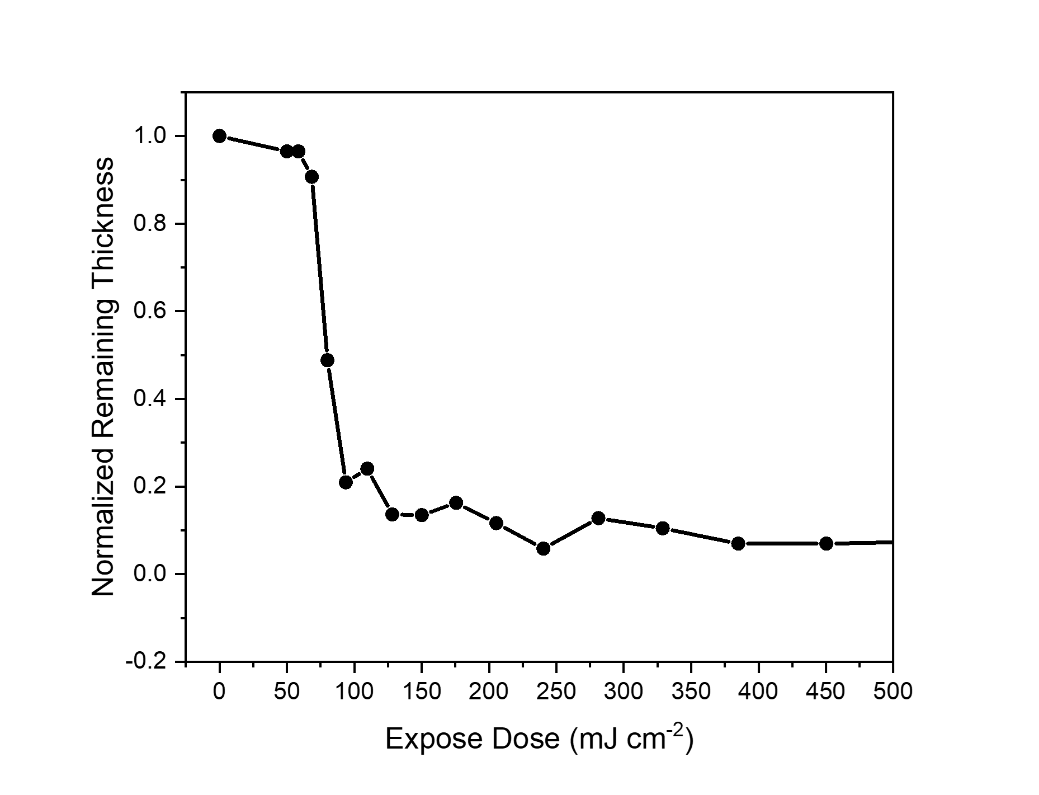


**Figure S3.** The sensitivity curves of ZIF-71 film at different EUV exposure doses.


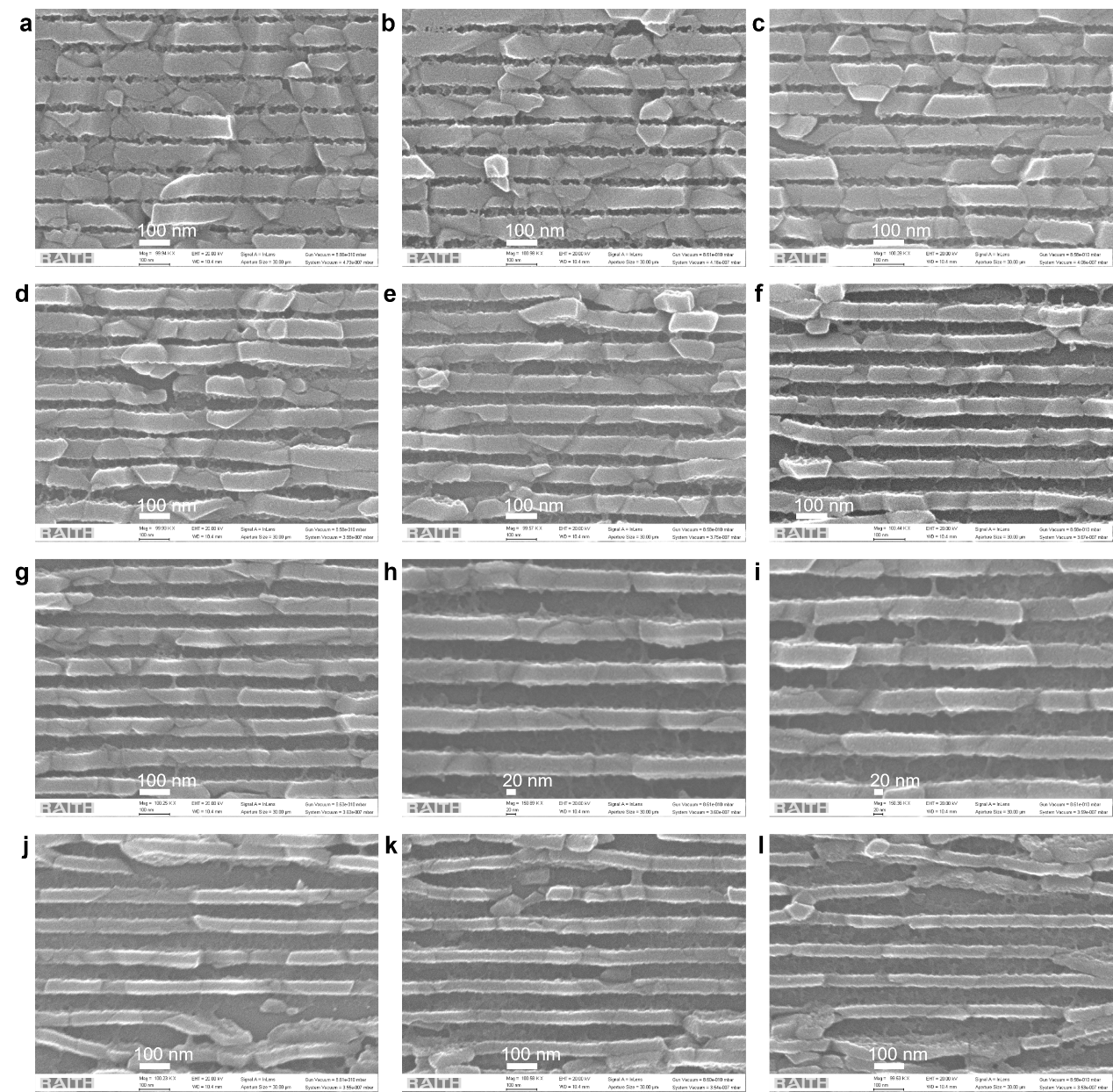


**Figure S4.** SEM images of the 50 nm HP patterns on 100 nm thick ZIF-71 films under various EUV exposure doses. (a) 250 mJ cm^−2^, (b) 270 mJ cm^−2^, (c) 300 mJ cm^−2^, (d) 330 mJ cm^−2^, (e) 360 mJ cm^−2^, (f) 400 mJ cm^−2^, (g) 440 mJ cm^−2^, (h) 480 mJ cm^−2^, (i) 530 mJ cm^−2^, (j) 580 mJ cm^−2^, (k) 640 mJ cm^−2^, (l) 705 mJ cm^−2^.


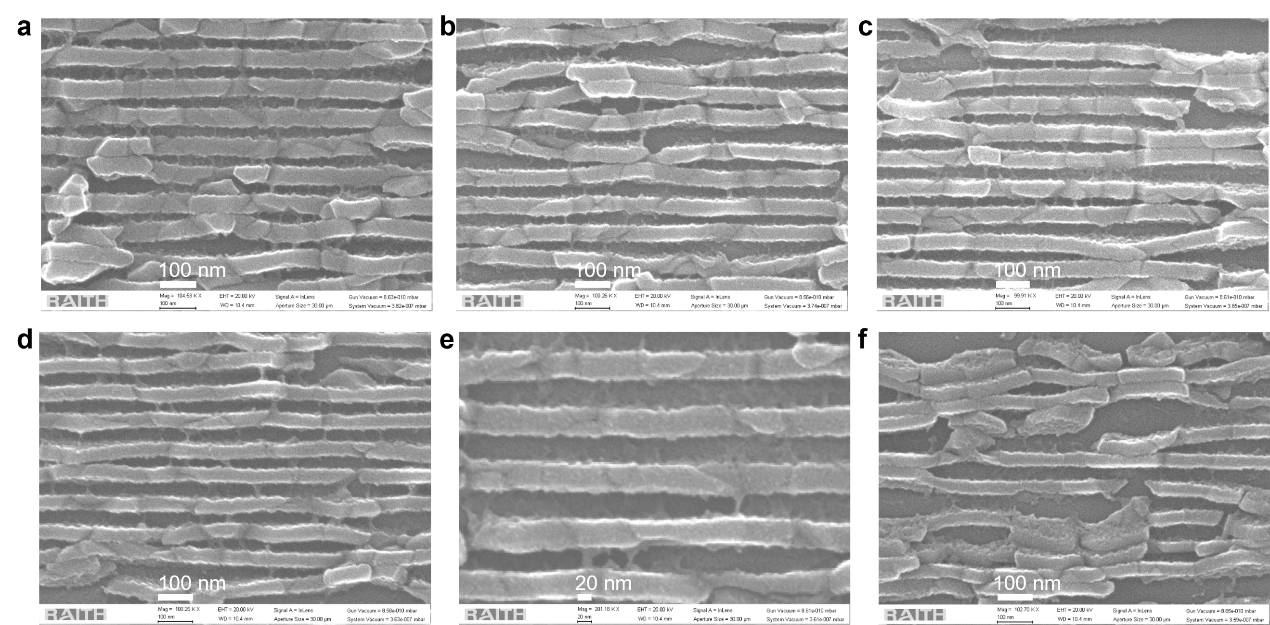


**Figure S5.** SEM images of the 40 nm HP patterns on 100 nm thick ZIF-71 films under various EUV exposure doses. (a) 240 mJ cm^−2^, (b) 265 mJ cm^−2^, (c) 290 mJ cm^−2^, (d) 320 mJ cm^−2^, (e) 350 mJ cm^−2^, (f) 385 mJ cm^−2^.


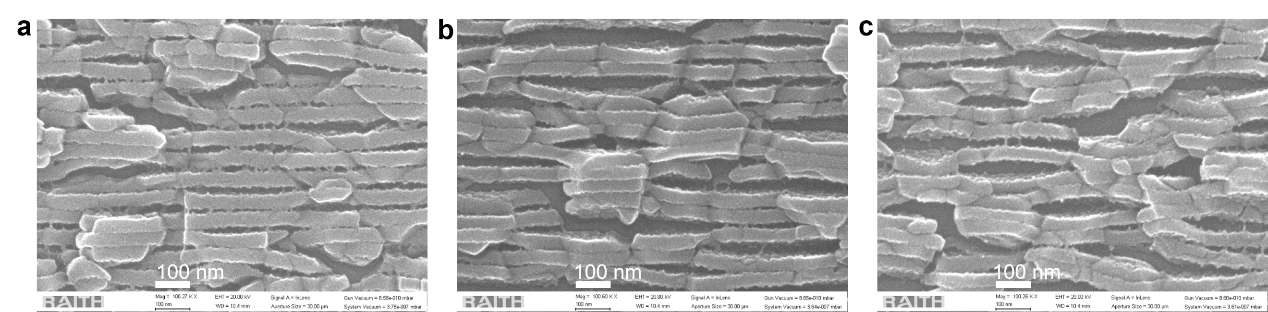


**Figure S6.** SEM images of the 30 nm HP patterns on 100 nm thick ZIF-71 films under various EUV exposure doses. (a) 250 mJ cm^−2^, (b) 280 mJ cm^−2^, (c) 305 mJ cm^−2^.


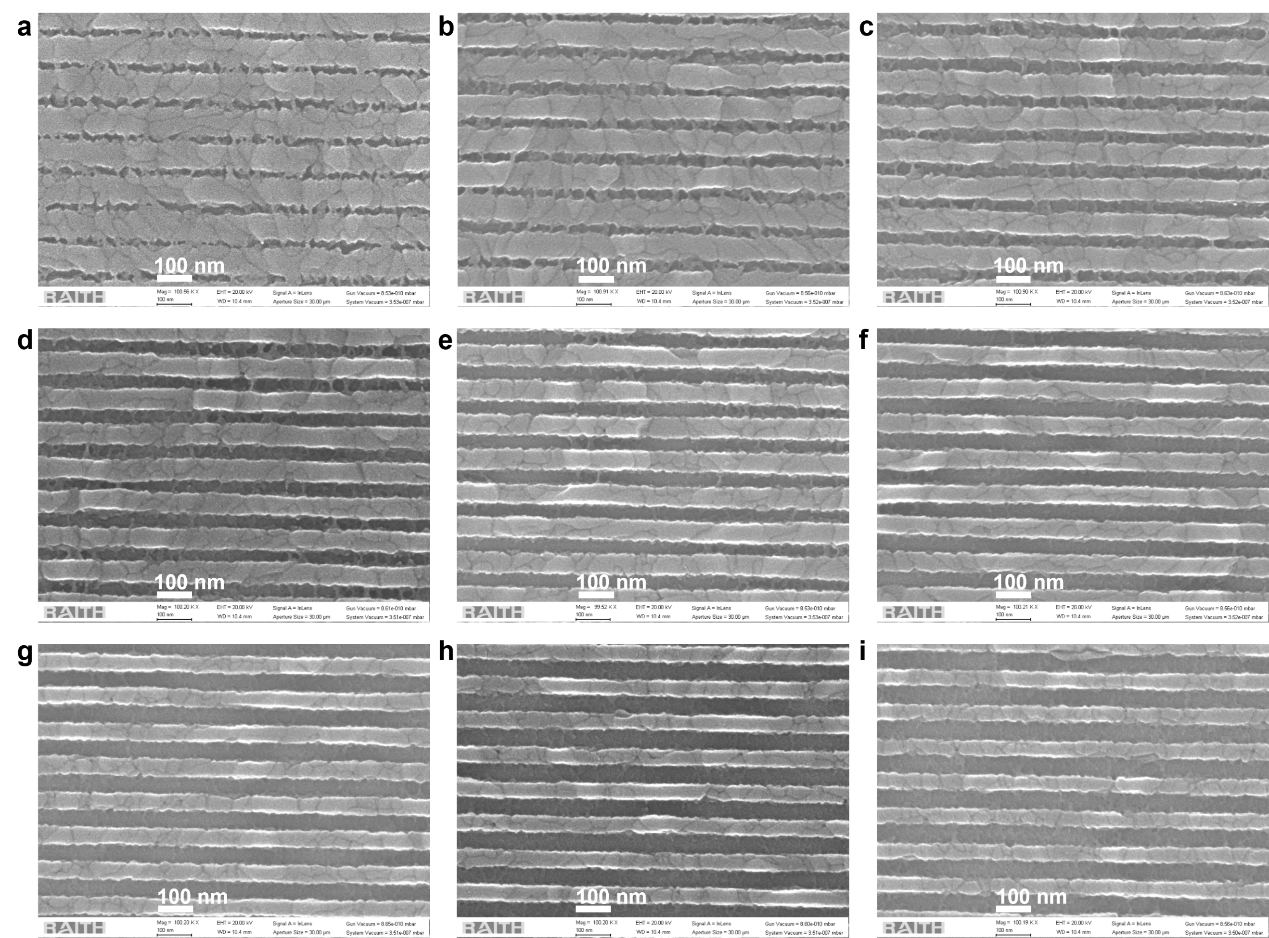


**Figure S7.** SEM images of the 50 nm HP patterns on 60 nm thick ZIF-71 films under various EUV exposure doses. (a) 250 mJ cm^−2^, (b) 270 mJ cm^−2^, (c) 300 mJ cm^−2^, (d) 330 mJ cm^−2^, (e) 360 mJ cm^−2^, (f) 400 mJ cm^−2^, (g) 440 mJ cm^−2^, (h) 480 mJ cm^−2^, (i) 530 mJ cm^−2^.

**
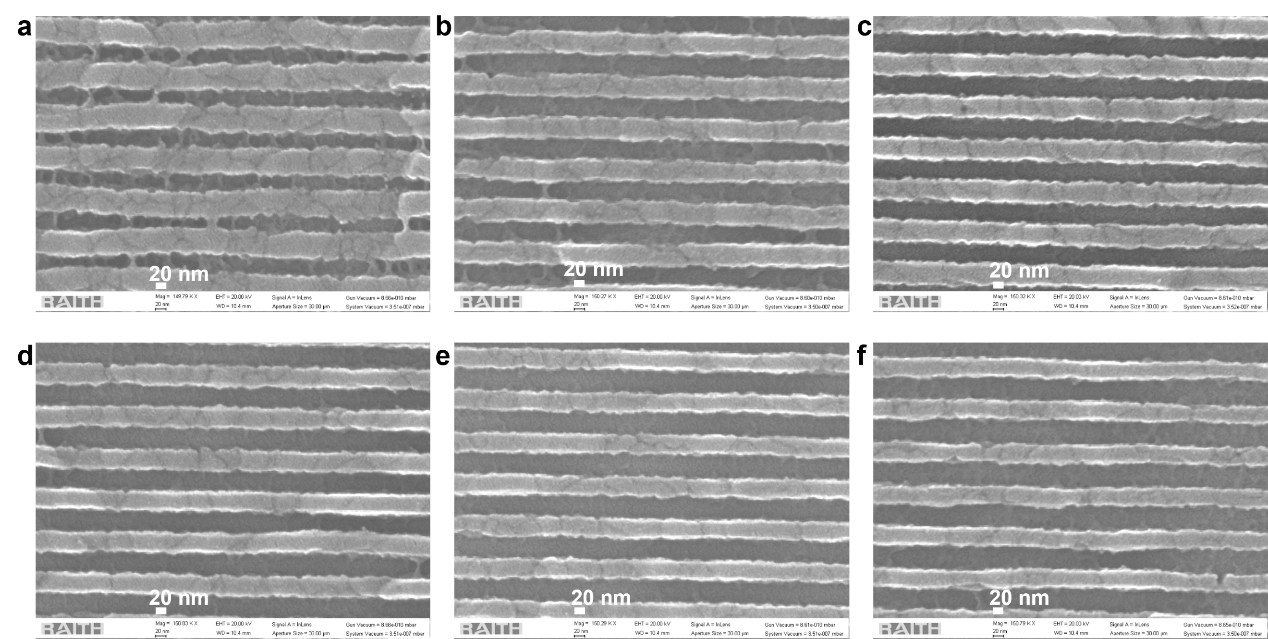
**

**Figure S8.** SEM images of the 40 nm HP patterns on 60 nm thick ZIF-71 films under various EUV exposure doses. (a) 240 mJ cm^−2^, (b) 320 mJ cm^−2^, (c) 385 mJ cm^−2^, (d) 425 mJ cm^−2^, (e) 465 mJ cm^−2^, (f) 515 mJ cm^−2^.

**
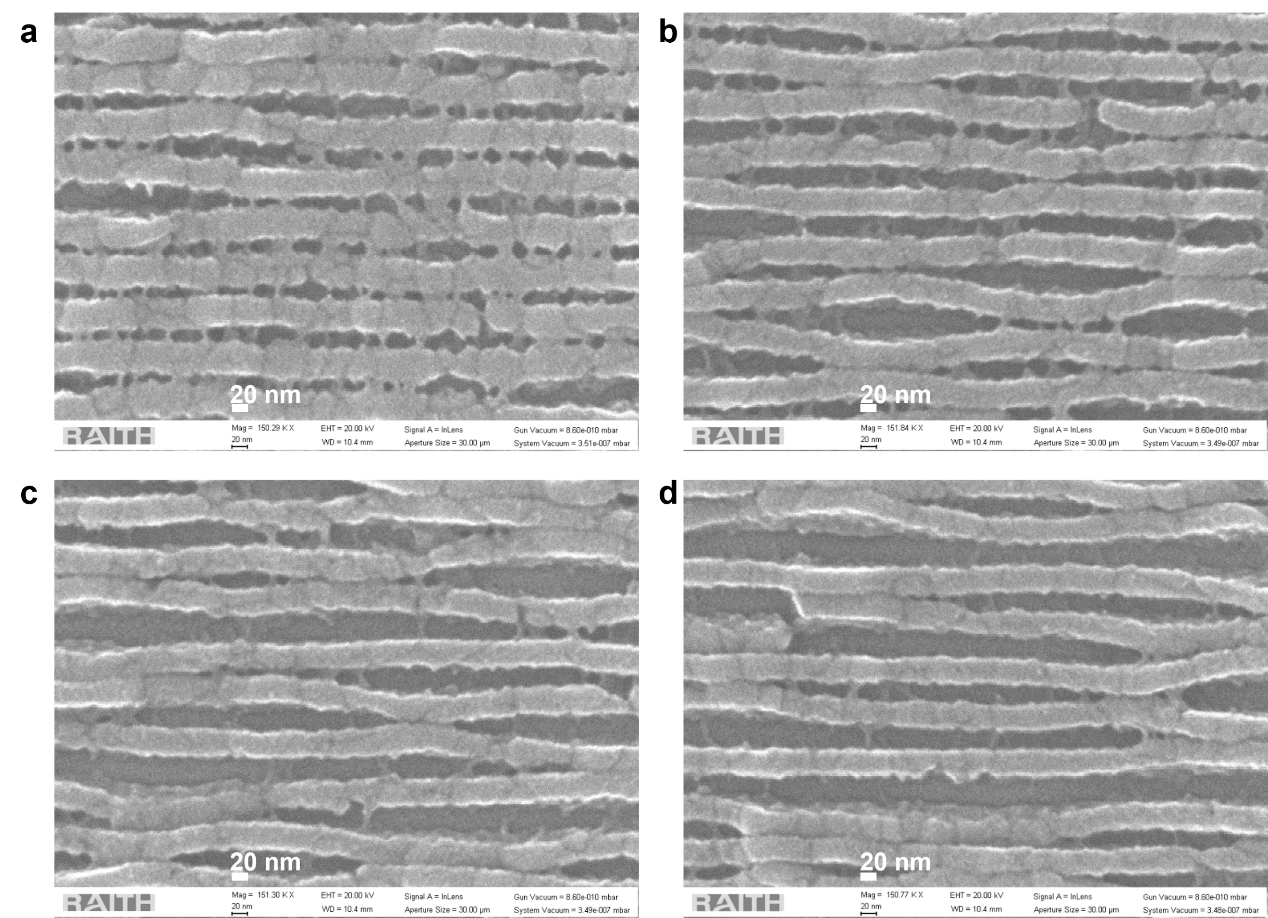
**

**Figure S9.** SEM images of the 30 nm HP patterns on 60 nm thick ZIF-71 films under various EUV exposure doses. (a) 250 mJ cm^−2^, (b) 280 mJ cm^−2^, (c) 305 mJ cm^−2^, (d) 335 mJ cm^−2^.

**
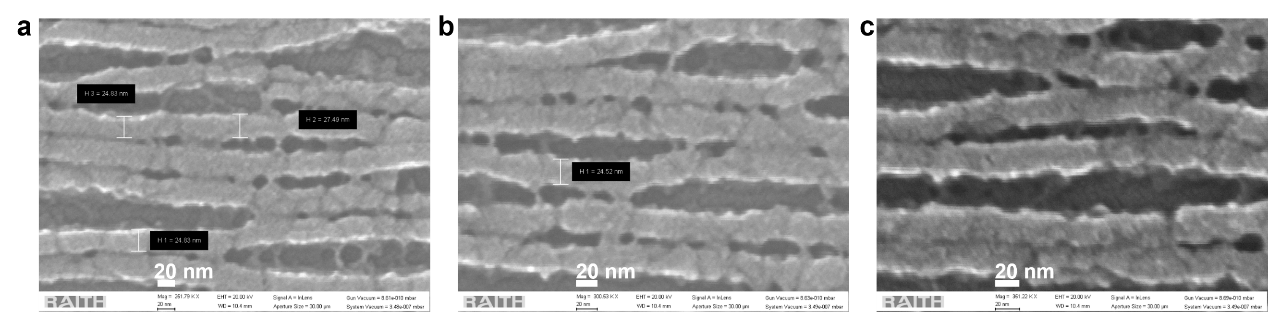
**

**Figure S10.** SEM images of the 22 nm HP patterns on 60 nm thick ZIF-71 films under various EUV exposure doses. (a) 240 mJ cm^−2^, (b) 265 mJ cm^−2^, (c) 290 mJ cm^−2^.

**
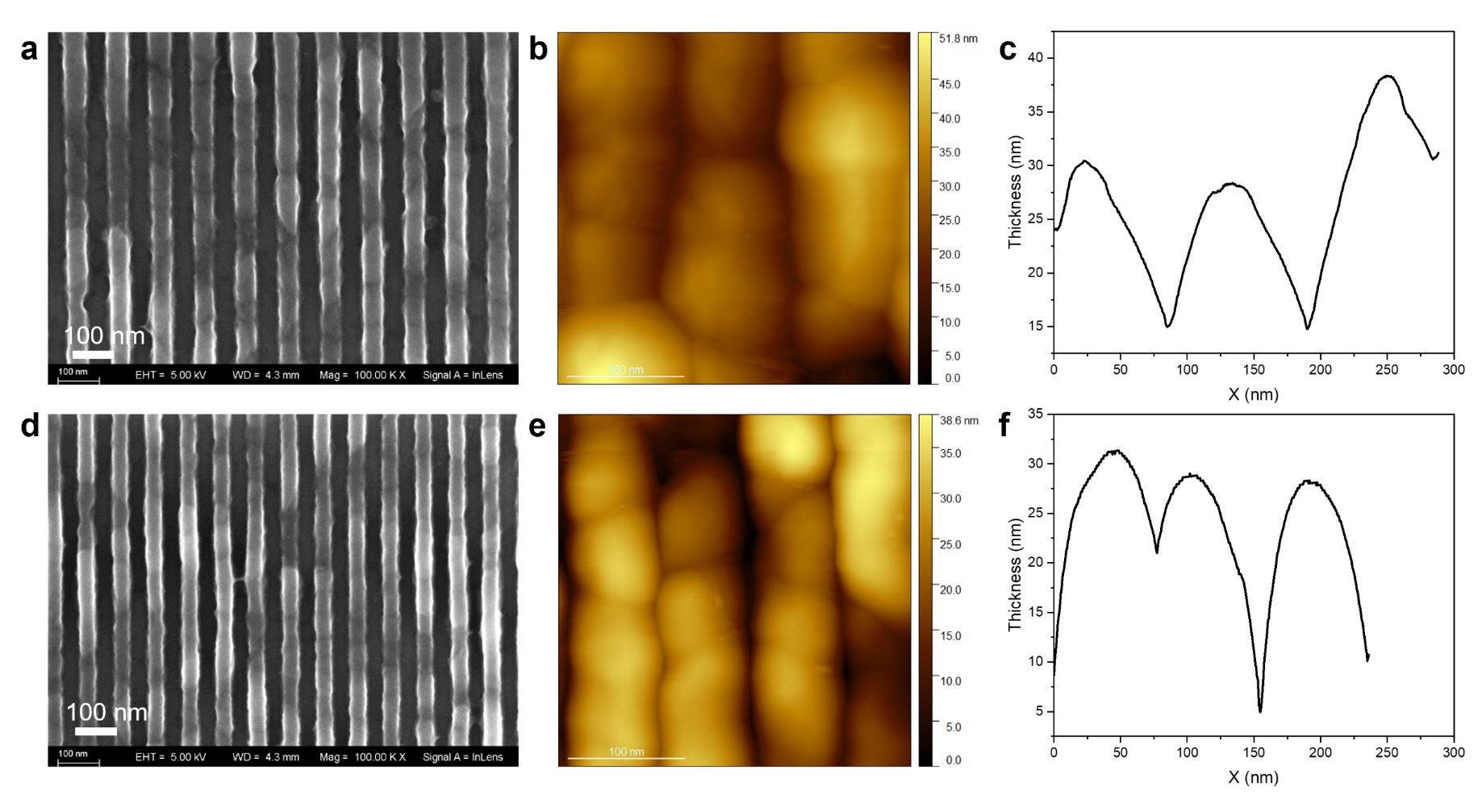
**

**Figure S11.** SEM, AFM images, and the section profiles of the AFM images of 60 nm thick ZIF-71 film with 50 nm HP (a, b, c), and 40 nm HP (d, e, f).

**
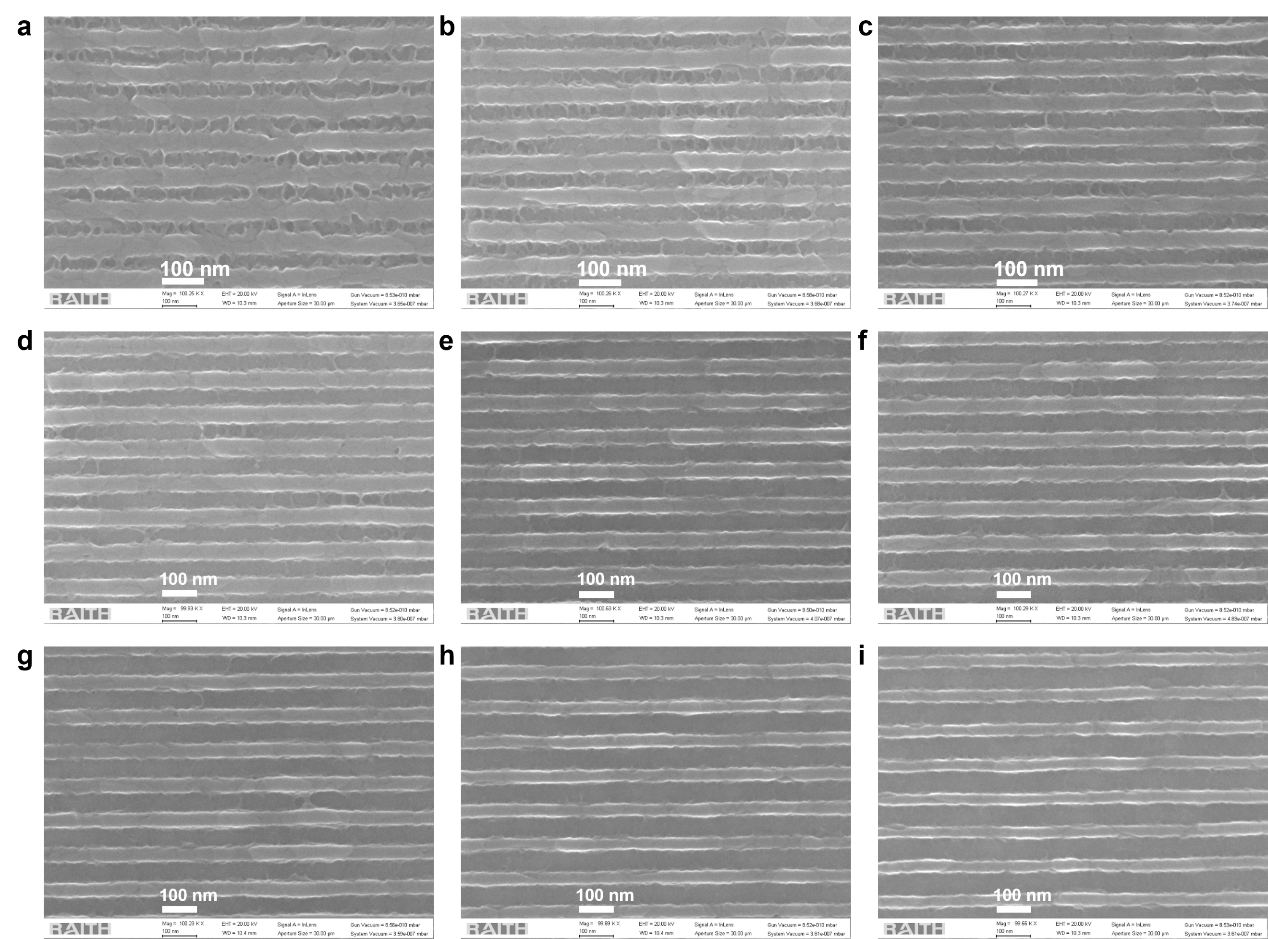
**

**Figure S12.** SEM images of the 50 nm HP patterns on 80 nm thick ZIF-8_Cl films under various EUV exposure doses. (a) 250 mJ cm^−2^, (b) 270 mJ cm^−2^, (c) 300 mJ cm^−2^, (d) 330 mJ cm^−2^, (e) 360 mJ cm^−2^, (f) 400 mJ cm^−2^, (g) 440 mJ cm^−2^, (h) 480 mJ cm^−2^, (i) 530 mJ cm^−2^.

**
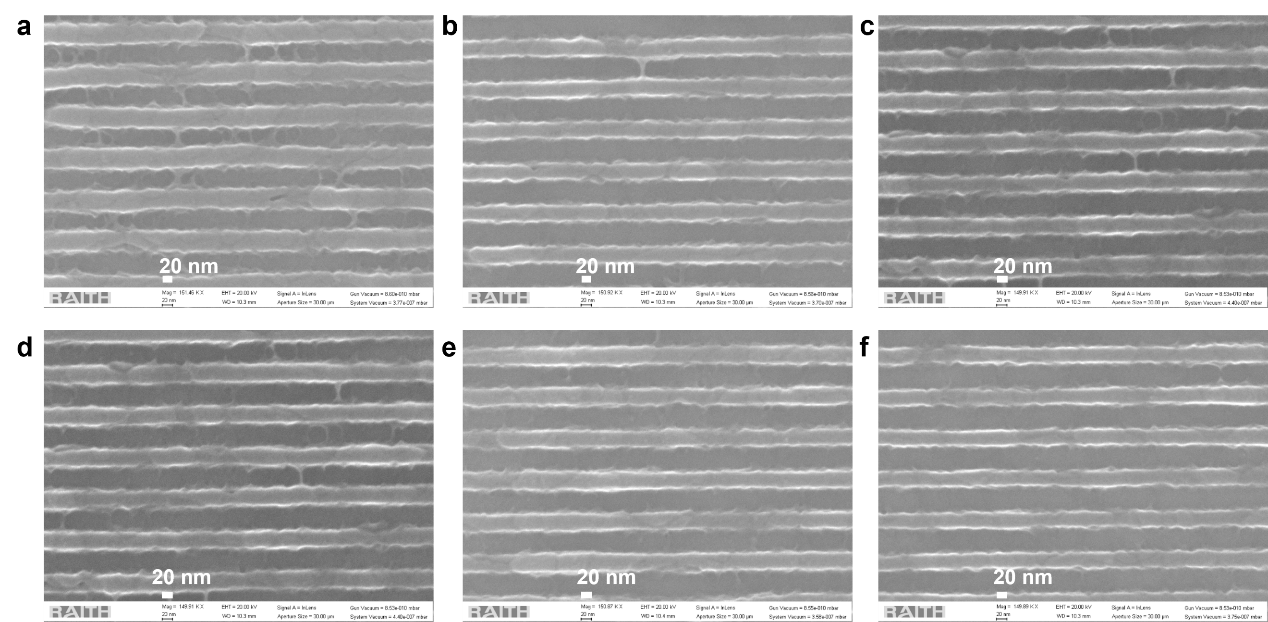
**

**Figure S13.** SEM images of the 40 nm HP patterns on 80 nm thick ZIF-8_Cl films under various EUV exposure doses. (a) 240 mJ cm^−2^, (b) 265 mJ cm^−2^, (c) 290 mJ cm^−2^, (d) 320 mJ cm^−2^, (e) 350 mJ cm^−2^, (f) 385 mJ cm^−2^.

**
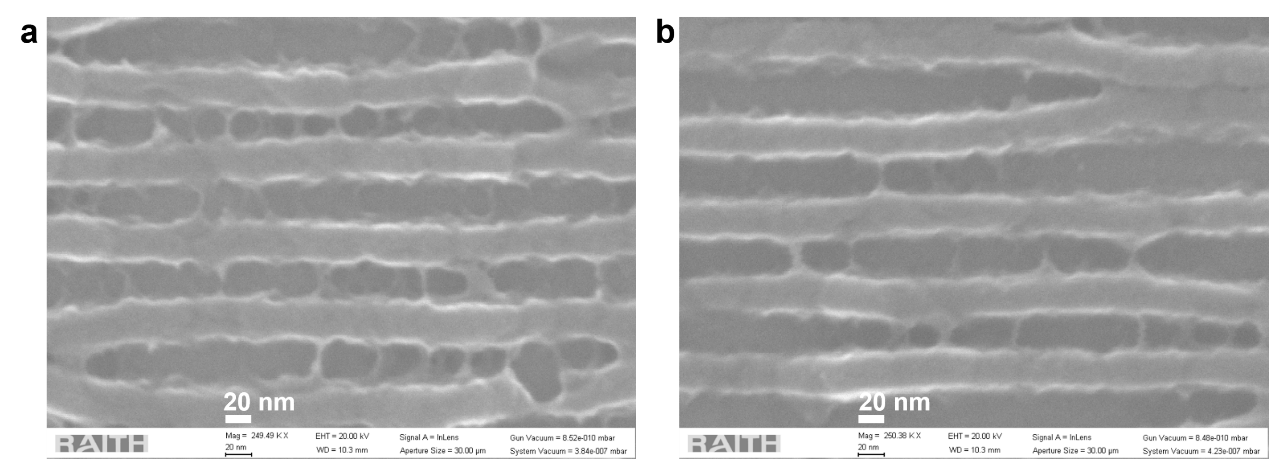
**

**Figure S14.** SEM images of the 30 nm HP patterns on 80 nm thick ZIF-8_Cl films under various EUV exposure doses. (a) 250 mJ cm^−2^, (b) 280mJ cm^−2^.

**
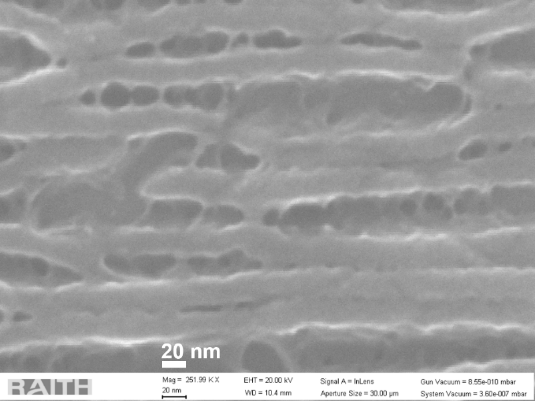
**

**Figure S15.** SEM images of the 22 nm HP patterns on 80 nm thick ZIF-8_Cl films under a EUV exposure dose of 240 mJ cm^−2^.

**
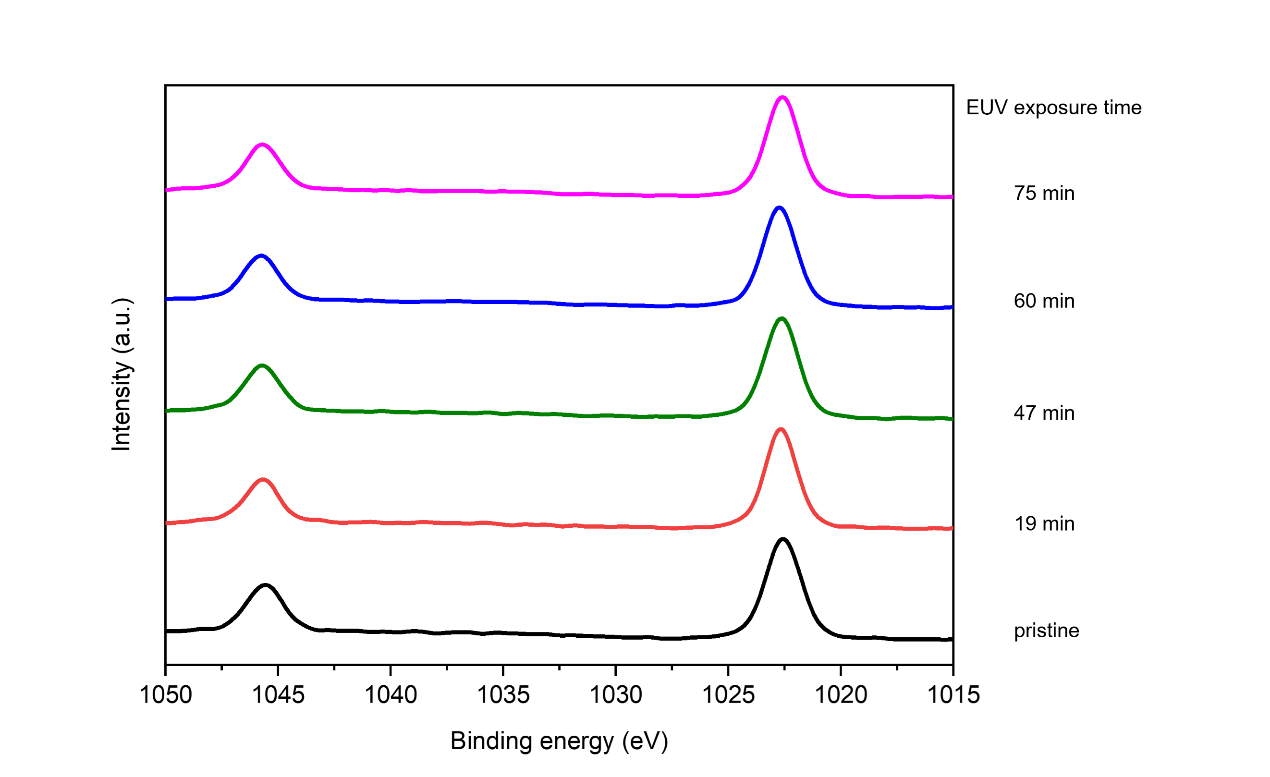
**

**Figure S16.** In situ XPS spectra of Zn 2p of ZIF-71 film under different EUV exposure times.

**
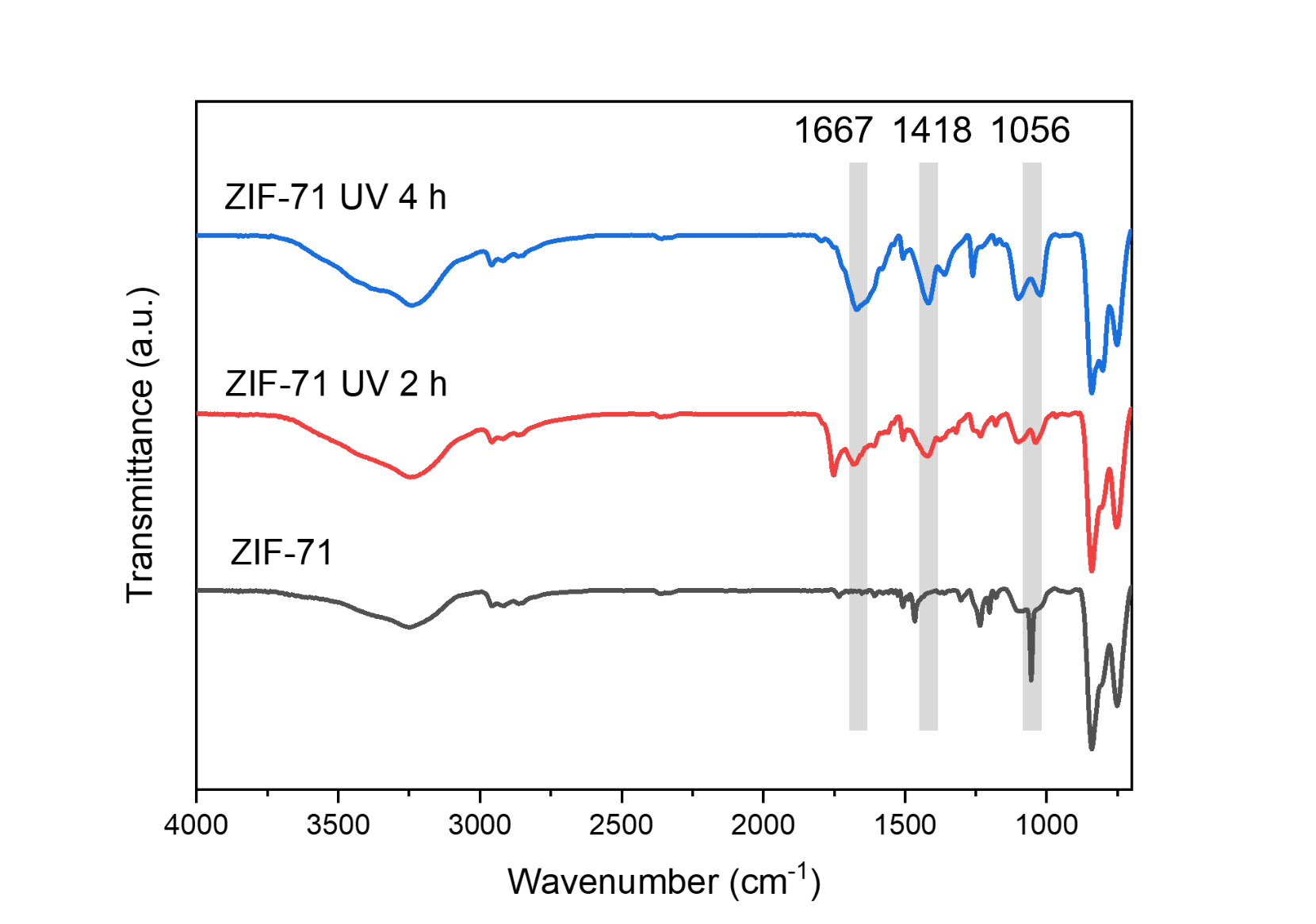
**

**Figure S17.** Infrared spectra of ZIF-71 film and ZIF-71 film exposed to UV at different times.

The open frame size for the EUV flood exposure is about 1.7 mm × 1.7 mm, which is not large enough for some spectroscopy sample preparation. Therefore, we recorded the IR spectra for ZIF-71 films exposed to 254 nm UV light, aiming for some indications of the chemical changes. FTIR spectroscopy was performed using a Bruker Vertex 70v spectrometer under vacuum at low temperature. After exposing ZIF-71 film to 254 nm UV light for 2 or 4 hours, its characteristic C-N vibration peak at 1056 cm^-1^ disappeared, while new peaks appeared at 1418 and 1667 cm^-1^ (C─H, N─H bending vibration), indicating the bond cleavage of the imidazolate linker and the formation of different imines, C-H bonds or amines.^[1–3]^

**
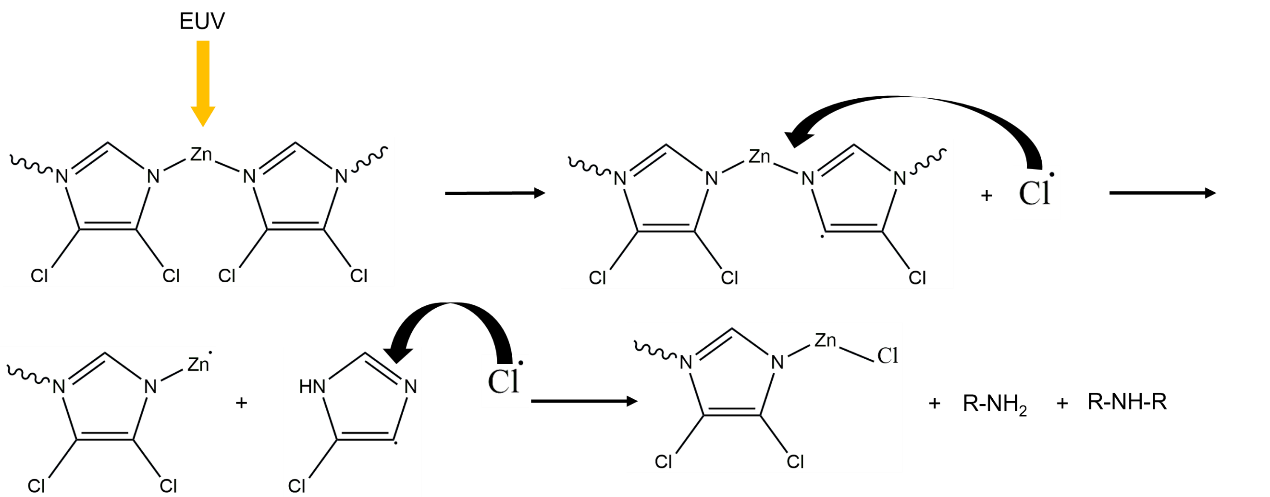
**

**Figure S18.** Proposed EUV-induced reactions on ZIF-71 film.


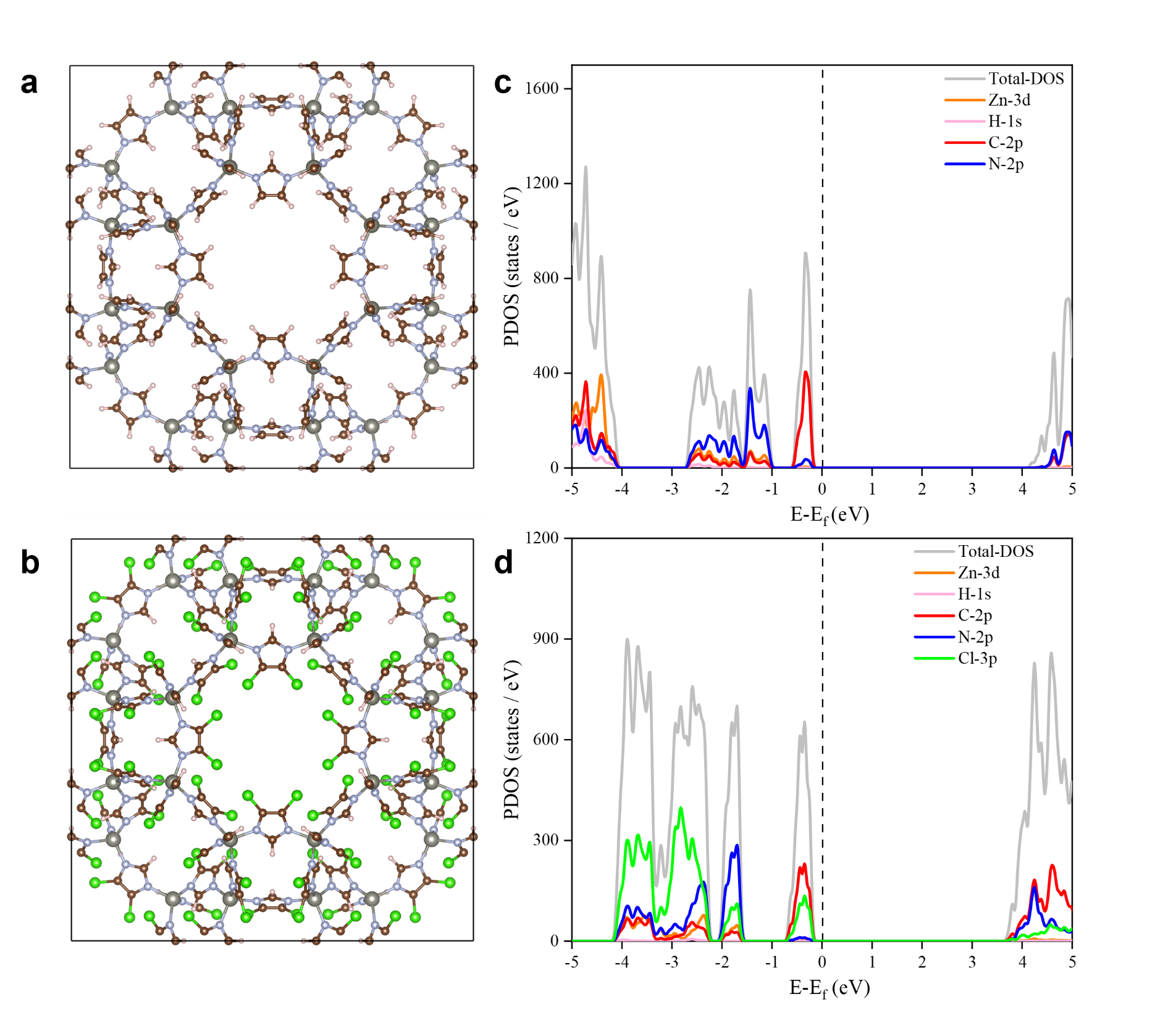


**Figure S19.** The crystal structure of (a) ZIF-71-H, and (b) ZIF-71-Cl, projected density of states of (c) ZIF-71-H, and (d) ZIF-71-Cl.

We performed all density functional theory (DFT) calculations using the Vienna Ab initio Simulation Package, applying the generalized gradient approximation with the Perdew-Burke-Ernzerh of functional.^[4,5,6]^ To describe the ionic cores, we used projected augmented wave potentials and included valence electrons with a plane-wave basis set and a kinetic energy cutoff of 400 eV.^[7,8]^ The Kohn-Sham orbitals were treated with partial occupancies using the Gaussian smearing method, with a width of 0.05 eV. For geometry and lattice size optimization, we performed Brillouin zone integration with 1 × 1 × 1 Γ-centered k-point sampling.^[9]^ Self-consistent calculations were conducted with an energy convergence threshold of 10^-5^ eV. Equilibrium geometries and lattice constants were optimized with a maximum stress of 0.02 eV Å^-1^ on each atom. Weak interactions were described using the DFT+D3 method, which includes empirical corrections based on Grimme’s scheme.^[10,11]^

The structure of ZIF-71-H is similar to that of ZIF-71-Cl, with hydrogen atoms replacing chlorine atoms. The bandgaps of ZIF-71-H and ZIF-71-Cl were 4.43 eV and 3.96 eV, respectively. Replacing hydrogen with chlorine reduces the bandgap, facilitating electron excitation to generate the photoelectrons and secondary electrons.^[12]^ This promotes the dissociative electron attachment reaction, enhancing the ZIF's photosensitivity. In addition, the replacement reduces the internal cohesion, thereby promoting the dissociation of weak bonds, which is favorable for enhancing the photosensitivity of the ZIFs.^[13]^

On the other hand, the halogenation enhances the absorption of EUV photons. The EUV energy of 92 eV is in the soft X-ray region. The X-ray attenuation length indicates the depth at which the intensity of X-rays decreases by 37% (1/e). It is determined by the material’s composition and density. A shorter attenuation length correlates with higher absorption. The EUV attenuation lengths for ZIF-71-H and ZIF-71-Cl were determined to be 0.30 μm and 0.23 μm, respectively, implying that halogenation enhances the absorption of EUV photons. This effect is beneficial for improving the photosensitivity of ZIFs.

**
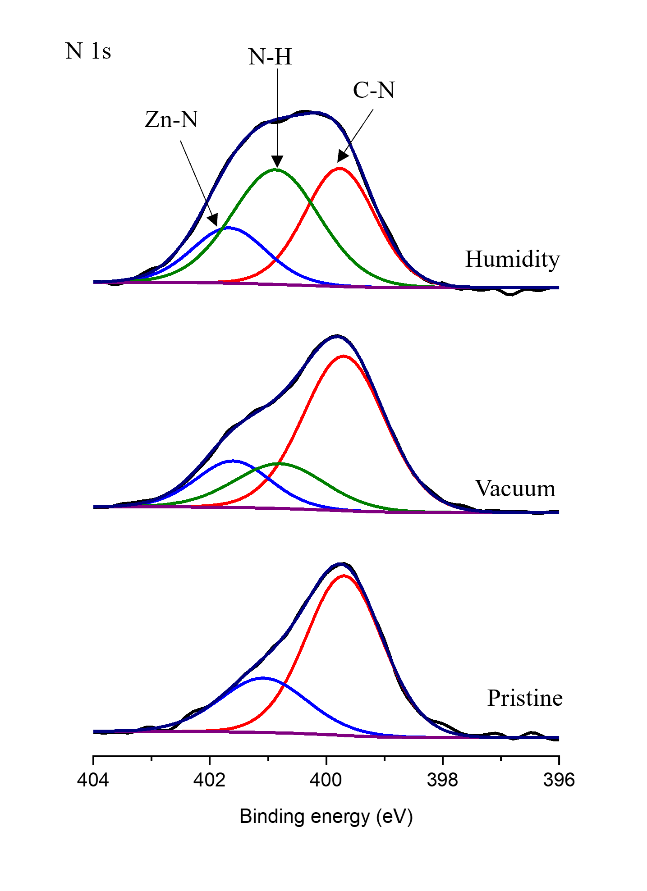
**

**Figure S20.** XPS spectra of N 1s exposed 60 min in different EUV exposure environments (exposure in a vacuum and humidity).

**
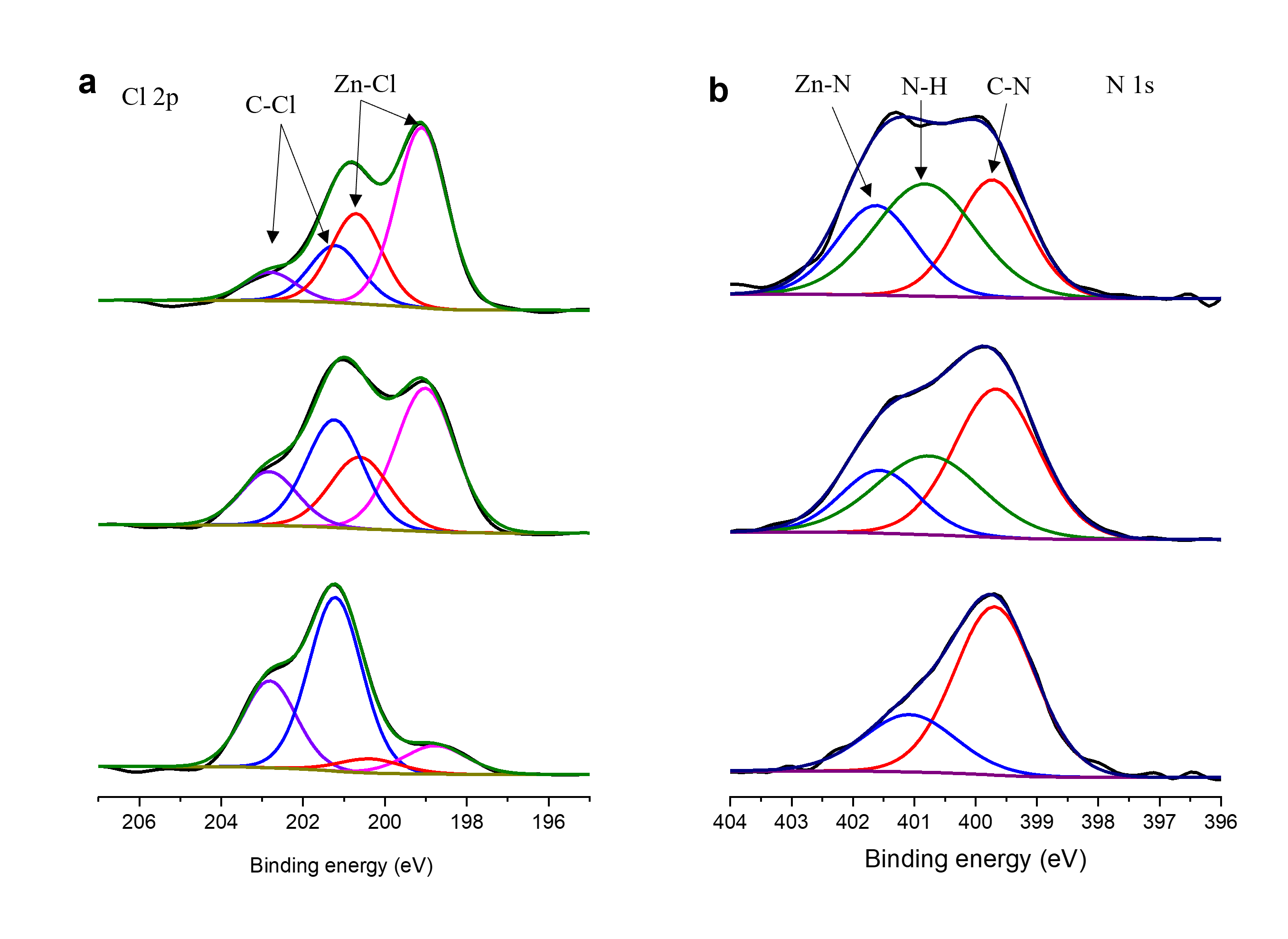
**

**Figure S21.** In situ XPS spectra of Cl 2p (a) and N 1s (b) of the ZIF-71 film, pristine (bottom), exposed 75 min in a vacuum (middle), and introducing a little water vapor at the same position after exposure (top).

**Table S1.** Linewidth of MOF pattern using various nano-patterning methods reported in the literature.

| Entry | MOF | Tone | Linewidth | Technique | Ref. |
| --- | --- | --- | --- | --- | --- |
| 1 | ZIF-8 | NA | 200 nm | Nanoimprinting | 14 |
| 2 | ZIF-71 | Positive | 40 nm | EBL | 15 |
| 3 | ZIF-8_Cl | Positive | 40 nm | EBL | 15 |
| 4 | ZIF-L | Negative | 30 nm | EBL | 16 |
| 5 | ZIF-8 | Positive | 150 nm | EBL | 17 |
| 6 | ZIF-8 | Negative | 20 nm | EBL | 18 |
| 7 | aZnMIm | Negative | 22 nm | EBL | 19 |
| 8 | HKUST-1 | Negative | 70 nm | EBL, using crosslinkers | 20 |
| 9 | HKUST-1 | NA | 50 nm | EUV, MOF grown on Patterned self-assembled monolayer | 21 |
| 10 | ZIF-71 | Positive | 40 nm | EUV | This work |
| 11 | ZIF-8_Cl | Positive | 40 nm | EUV | This work |

**References**

[1] S. D. N. P., S. S. S., S. C., J. D. S., S. P., *JASR* **2021**, *12*, 176.

[2] Y. Li, L. H. Wee, J. A. Martens, I. F. J. Vankelecom, *J. Mater. Chem. A* **2014**, *2*, 10034.

[3] H. Yin, P. Cay-Durgun, T. Lai, G. Zhu, K. Engebretson, R. Setiadji, M. D. Green, M. L. Lind, *Polymer* **2020**, *195*, 122379.

[4] G. Kresse, J. Furthmüller, *Comput. Mater. Sci.* **1996**, *6*, 15.

[5] G. Kresse, J. Furthmüller, *Phys. Rev. B* **1996**, *54*, 11169.

[6] J. P. Perdew, K. Burke, M. Ernzerhof, *Phys. Rev. Lett.* **1996**, *77*, 3865.

[7] G. Kresse, D. Joubert, *Phys. Rev. B* **1999**, *59*, 1758.

[8] P. E. Blöchl, *Phys. Rev. B* **1994**, *50*, 17953.

[9] H. J. Monkhorst, J. D. Pack, *Phys. Rev. B* **1976**, *13*, 5188.

[10] S. Grimme, J. Antony, S. Ehrlich, H. Krieg, *J. Chem. Phys.* **2010**, *132*, 154104.

[11] S. Grimme, S. Ehrlich, L. Goerigk, *J. Comput. Chem.* **2011**, *32*, 1456.

[12] J. Edzards, H.-D. Saßnick, A. G. Buzanich, A. M. Valencia, F. Emmerling, S. Beyer, C. Cocchi, *J. Phys. Chem. C* **2023**, *127*, 21456.

[13] M. Xiong, N. Li, G. Yin, W.-Y. Ching, X. Zhao, *J. Non. Cryst. Solids* **2020**, *536*, 120005.

[14] O. Dalstein, D. R. Ceratti, C. Boissière, D. Grosso, A. Cattoni, M. Faustini, *Adv. Funct. Mater.* **2016**, *26*, 81.

[15] M. Tu, B. Xia, D. E. Kravchenko, M. L. Tietze, A. J. Cruz, I. Stassen, T. Hauffman, J. Teyssandier, S. De Feyter, Z. Wang, R. A. Fischer, B. Marmiroli, H. Amenitsch, A. Torvisco, M. de J. Velásquez-Hernández, P. Falcaro, R. Ameloot, *Nat. Mater.* **2021**, *20*, 93.

[16] Y. Miao, M. Tsapatsis, *Chem. Mater.* **2021**, *33*, 754.

[17] Y. Miao, D. T. Lee, M. D. De Mello, M. Ahmad, M. K. Abdel-Rahman, P. M. Eckhert, J. A. Boscoboinik, D. H. Fairbrother, M. Tsapatsis, *Nat. Commun.* **2022**, *13*, 420.

[18] Q. Liu, Y. Miao, L. F. Villalobos, S. Li, H.-Y. Chi, C. Chen, M. T. Vahdat, S. Song, D. J. Babu, J. Hao, Y. Han, M. Tsapatsis, K. V. Agrawal, *Nat. Mater.* **2023**, *22*, 1387.

[19] P. Corkery, K. E. Waltz, P. M. Eckhert, M. Ahmad, A. Kraetz, Y. Miao, D. T. Lee, M. K. Abdel-Rahman, Y. Lan, P. Haghi-Ashtiani, A. Stein, J. A. Boscoboinik, M. Tsapatsis, D. H. Fairbrother, *Adv. Funct. Mater.* **2024**, *34*, 2311149.

[20] X. Tian, F. Li, Z. Tang, S. Wang, K. Weng, D. Liu, S. Lu, W. Liu, Z. Fu, W. Li, H. Qiu, M. Tu, H. Zhang, J. Li, *Nat. Commun.* **2024**, *15*, 2920.

[21] O. Lugier, N. Thakur, L. Wu, M. Vockenhuber, Y. Ekinci, S. Castellanos, *ACS Appl. Mater. Interfaces* **2021**, *13*, 43777.
